# Assessing trends in the content of maternal and child care following a health system strengthening initiative in rural Madagascar: A longitudinal cohort study

**DOI:** 10.1371/journal.pmed.1002869

**Published:** 2019-08-20

**Authors:** Camille Ezran, Matthew H. Bonds, Ann C. Miller, Laura F. Cordier, Justin Haruna, David Mwanawabenea, Marius Randriamanambintsoa, Hery-Tiana R. Razanadrakato, Mohammed Ali Ouenzar, Bénédicte R. Razafinjato, Megan Murray, Andres Garchitorena

**Affiliations:** 1 Department of Health Research and Policy, Stanford University School of Medicine, Stanford, California, United States of America; 2 PIVOT, Ranomafana, Madagascar; 3 Department of Global Health and Social Medicine, Harvard Medical School, Blavatnik Institute, Boston, Massachusetts, United States of America; 4 Direction de la Démographie et des Statistiques Sociales, Institut National de la Statistique, Antananarivo, Madagascar; 5 MIVEGEC, Univ Montpellier, CNRS, IRD, Montpellier, France; London School of Hygiene and Tropical Medicine, UNITED KINGDOM

## Abstract

**Background:**

In order to reach the health-related Sustainable Development Goals (SDGs) by 2030, gains attained in access to primary healthcare must be matched by gains in the quality of services delivered. Despite the broad consensus around the need to address quality, studies on the impact of health system strengthening (HSS) have focused predominantly on measures of healthcare access. Here, we examine changes in the content of maternal and child care as a proxy for healthcare quality, to better evaluate the effectiveness of an HSS intervention in a rural district of Madagascar. The intervention aimed at improving system readiness at all levels of care (community health, primary health centers, district hospital) through facility renovations, staffing, equipment, and training, while removing logistical and financial barriers to medical care (e.g., ambulance network and user-fee exemptions).

**Methods and findings:**

We carried out a district-representative open longitudinal cohort study, with surveys administered to 1,522 households in the Ifanadiana district of Madagascar at the start of the HSS intervention in 2014, and again to 1,514 households in 2016. We examined changes in healthcare seeking behavior and outputs for sick-child care among children <5 years old, as well as for antenatal care and perinatal care among women aged 15–49. We used a difference-in-differences (DiD) analysis to compare trends between the intervention group (i.e., people living inside the HSS catchment area) and the non-intervention comparison group (i.e., the rest of the district). In addition, we used health facility–based surveys, monitoring service availability and readiness, to assess changes in the operational capacities of facilities supported by the intervention. The cohort study included 657 and 411 children (mean age = 2 years) reported to be ill in the 2014 and 2016 surveys, respectively (27.8% and 23.8% in the intervention group for each survey), as well as 552 and 524 women (mean age = 28 years) reported to have a live birth within the previous two years in the 2014 and 2016 surveys, respectively (31.5% and 29.6% in the intervention group for each survey). Over the two-year study period, the proportion of people who reported seeking care at health facilities experienced a relative change of +51.2% (from 41.4% in 2014 to 62.5% in 2016) and −7.1% (from 30.0% to 27.9%) in the intervention and non-intervention groups, respectively, for sick-child care (DiD *p*-value = 0.01); +11.4% (from 78.3% to 87.2%), and +10.3% (from 67.3% to 74.2%) for antenatal care (*p*-value = 0.75); and +66.2% (from 23.1% to 38.3%) and +28.9% (from 13.9% to 17.9%) for perinatal care (*p*-value = 0.13). Most indicators of care content, including rates of medication prescription and diagnostic test administration, appeared to increase more in the intervention compared to in the non-intervention group for the three areas of care we assessed. The reported prescription rate for oral rehydration therapy among children with diarrhea changed by +68.5% (from 29.6% to 49.9%) and −23.2% (from 17.8% to 13.7%) in the intervention and non-intervention groups, respectively (*p*-value = 0.05). However, trends observed in the care content varied widely by indicator and did not always match the large apparent increases observed in care seeking behavior, particularly for antenatal care, reflecting important gaps in the provision of essential health services for individuals who sought care. The main limitation of this study is that the intervention catchment was not randomly allocated, and some demographic indicators were better for this group at baseline than for the rest of the district, which could have impacted the trends observed.

**Conclusion:**

Using a district-representative longitudinal cohort to assess the content of care delivered to the population, we found a substantial increase over the two-year study period in the prescription rate for ill children and in all World Health Organization (WHO)-recommended perinatal care outputs assessed in the intervention group, with more modest changes observed in the non-intervention group. Despite improvements associated with the HSS intervention, this study highlights the need for further quality improvement in certain areas of the district’s healthcare system. We show how content of care, measured through standard population-based surveys, can be used as a component of HSS impact evaluations, enabling healthcare leaders to track progress as well as identify and address specific gaps in the provision of services that extend beyond care access.

## Abstract 2

Camille Ezran and colleagues reveal changes in the content of maternal and child care as a proxy for wider changes in healthcare in general in Madagascar.

## Author summary

**Why was this study done?:**

- One of the largest contributors to preventable deaths in low- and middle-income countries is poor quality of care delivered by the public health system. Yet, given the multiple dimensions of care quality, there are currently no standard measures to evaluate impacts in this domain.
- While health system strengthening (HSS) initiatives strive to simultaneously increase access to healthcare and improve care quality, few studies have evaluated the impact of HSS initiatives on both targets. Our study sought to determine whether an HSS intervention in a rural health district of Madagascar, one of the world’s poorest countries, improved the quality of maternal and child care at the population level.

**What did the researchers do and find?:**

- We used data from a district-representative open longitudinal cohort that followed over 1,500 households between 2014 and 2016, in order to compare trends in the content of care as a proxy for care quality, inside and outside the intervention catchment, through difference-in-differences analyses. We also analysed data from a Service Availability and Readiness Assessment (SARA) conducted in health facilities supported by the HSS intervention.
- Our data set included self-reported information on health seeking behaviors and care content for common illnesses of children under five (*n* = 657 in 2014; 411 in 2016), and for maternal care before and during live births that occurred in the previous two years (*n* = 552 in 2014; 524 in 2016).
- We found that compared to the non-intervention group, the intervention group experienced a larger improvement in most care content outputs for childhood illnesses (e.g., 24.4% more children with diarrhea were prescribed oral rehydration therapy after two years than in the non-intervention area) and for perinatal care, whereas trends in antenatal care content were more similar in both populations.
- Despite progress, there remained important gaps in the provision of essential health services for individuals in both the intervention and non-intervention groups.

**What do these findings mean?:**

- The study provides evidence that HSS initiatives can successfully increase access to healthcare in target populations while also improving the quality of certain primary care services provided.
- The approach used here can be adapted to other local HSS initiatives to stimulate more comprehensive impact evaluations.
- Evidence from this study can help guide investments in integrated primary care systems that are needed globally to improve maternal and child care.
- The interpretation of the study findings is limited by the absence of randomization in the allocation of the intervention’s programs and by the reliance on self-reported answers.

## Introduction

The advent of the United Nations Sustainable Development Goals (SDGs) in 2015 bolstered a global commitment towards achieving universal health coverage (UHC) for all populations by 2030 through strengthened primary care [1,2]. As the 40th anniversary of the Declaration of Alma-Ata for UHC was recently celebrated [3], an estimated 60% of the world’s population has access to quality essential healthcare services, medicines, and vaccines, as well as financial risk protection [4]. Although achieving UHC for the remaining approximately 3 billion people in the next decade will require substantial investment [5], recent progress in several low- and middle-income countries such as Cambodia and Rwanda suggests that this ambitious goal is within reach [6]. Their gains in health coverage have been attributed in large part to reductions in financial barriers and sustained investments to strengthen the health system across the entire continuum of care [7,8].

In an effort to promote evidence-based models of health system strengthening (HSS) that can be scaled up nationwide and across borders, the global health community has urged for more rigorous evaluation of the impact of such interventions [9,10]. While past studies evaluating HSS initiatives predominantly focused on measures of access to healthcare (“care seeking behavior” or “coverage”), it is now evident that improving access alone is not sufficient to achieve the health-related SDGs [11–13]. There is growing recognition that measuring care quality is necessary to more comprehensively assess an intervention’s effectiveness in improving population health outcomes [14–17].

The Lancet Global Health Commission on High Quality Health Systems defines 10 essential components of high-quality health systems, including better health outcomes, competent care, positive user experience, and governance (**S1 Appendix**) [10]. Given the number of contributors to effective healthcare, there are dozens of possible measurable outcomes to evaluate care quality impacts of HSS interventions, ranging from measures of population health outcomes (e.g., disease incidence rates, mortality rates) to more targeted indicators (e.g., care provider performance assessments, vaccination rates) [18–21]. However, such indicators of care quality currently lack standardization, limiting the ability to compare across studies and produce generalizable conclusions [10,22].

The term “content of care” refers to the activity outputs of an intervention, including the medications prescribed, diagnostic tests performed, and counseling provided to patients [18]. Determining the rate of healthcare outputs, such as the provision of recommended diagnoses and treatments, helps assess an aspect of care quality (competent care) in a manner that is quantifiable, objective, and applicable to a variety of diseases and conditions. While there have been increasing efforts in recent years to understand content of care in low resource settings [23–26], the integration of this measure in impact evaluations of HSS interventions using population-level data remains scarce [7].

We evaluate the impact of an HSS intervention on the content of care delivered to a target population in rural Madagascar. Madagascar is one of the poorest countries, with among the lowest per capita healthcare spending in the world [27]. Consequently, the public health system lacks resources to ensure appropriate service provision to its population of 27 million. As of 2012, there were approximately 3.6 physicians, nurses, and midwives per 10,000 people [28], one tenth of the minimal threshold density (34.5 per 10,000) considered necessary to achieve high coverage for essential health services [29]. That same year, the national maternal, under-five, and neonatal mortality rates were 478/100,000 live births, 62/1,000, and 26/1,000, respectively [30]. In 2014, the nongovernmental organization PIVOT partnered with Madagascar’s Ministry of Health (MoH) to create a model health system within the government district of Ifanadiana through strengthening the existing public healthcare system with improving facility readiness, clinical programs, and integrated data systems at all levels of care [31,32].

We build on a previously published evaluation that revealed rapid increases in care seeking behavior to health centers and decreases in neonatal and under-five mortality rates associated with the HSS intervention [33]. Using the same district-representative longitudinal cohort study [34], we examine changes in the content of care provided to the population over the same period. We focus on three areas of healthcare that have a demonstrated impact on maternal and child mortality rates: sick-child care (<5 years old), antenatal care, and perinatal care [35,36]. In addition, we assess changes in the level of service availability (the physical presence of services) and readiness (the components required to provide services)—measures necessary for the provision of quality care [37]—in primary health centers supported by the PIVOT-MoH initiative. By evaluating changes in content of care at the population level, we thus deepen our understanding of the impact of an HSS intervention in a way that weighs accessibility and quality as complementary components of effective healthcare delivery.

## Methods

### Study intervention

**Fig 1:**
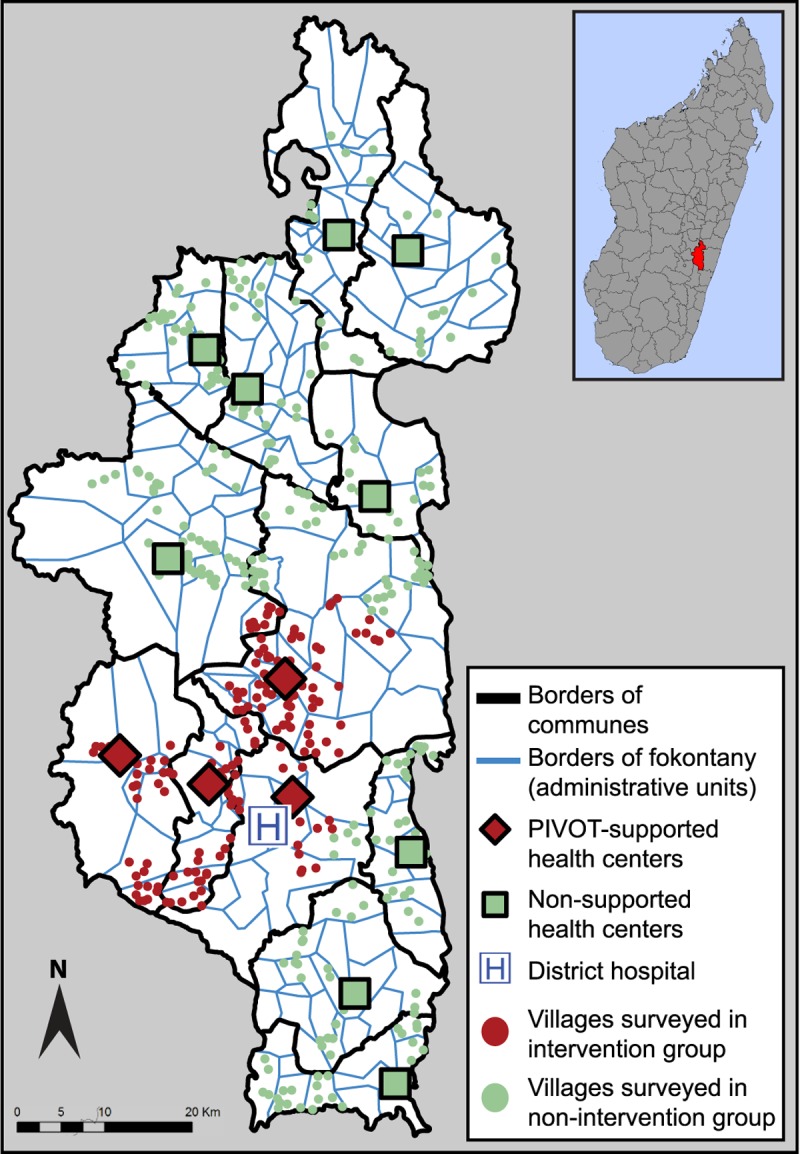
Map of the Ifanadiana district in southeastern Madagascar. Ifanadiana has an estimated population of 200,000 people, approximately one third of which live within PIVOT’s initial catchment area. The district is comprised of 13 communes (demarcated by black lines) and 195 fokontany (the smallest administrative unit, demarcated by blue lines). Each of the communes contains a primary health center (Centre de Santé de Base 2: red diamonds and green squares). Between 2014 and 2016, PIVOT and the MoH renovated, staffed, and equipped four of them (red diamonds). The households surveyed by the PIVOT-MoH longitudinal cohort study that were located in villages nearest to a PIVOT-supported health center were categorized into the intervention group (red dots); the households located in villages nearest to a nonsupported health center were categorized into the non-intervention group (green dots). Map of Madagascar in the top right corner, with the Ifanadiana district colored in red. Base maps obtained from INSTAT and GADM.org. INSTAT, National Institute of Statistics; MoH, Ministry of Health.

Since 2014, PIVOT and Madagascar’s MoH have collaborated to design, implement, and assess an HSS intervention in the southeastern district of Ifanadiana (**Fig 1**) as a model healthcare delivery system for the country [31,32]. In the first two years of the intervention, the PIVOT-MoH partnership primarily focused on a catchment area that included 4 out of the district’s 13 communes, encompassing approximately one third of the 200,000 people living in Ifanadiana. This initial intervention area contains the district’s sole hospital and four of its primary health centers. The choice of the catchment area was done according to logistical and programmatic reasons, and there was no randomization of communes involved. Guided by the World Health Organization’s (WHO) framework for functional HSS [9], the partnership targeted all three levels of care governed by the MoH in the catchment area (community health, primary health centers, district hospital) [32]. In brief, to improve readiness, PIVOT-MoH renovated, staffed, and equipped the hospital and health centers located in the catchment area since mid-2014, as well as initiated a community health program in a subset of the catchment’s remote villages by November 2015. In addition, the partnership sought to remove logistical and financial barriers to medical care by creating an ambulance network and removing fees for commonly prescribed medications for all patients [38]. PIVOT-MoH also implemented WHO’s Integrated Management of Childhood Illness (IMCI) guidelines [39] and national guidelines for the treatment for severe acute malnutrition, as well as had social workers at health facilities for the accompaniment and follow-up of vulnerable patients. Details on the intervention are available in S1 TIDIER Checklist using the Template for Intervention Description and Replication (TIDieR) [40].

During this period, two independent groups implemented complementary health programs in the Ifanadiana district. The World Bank–funded Emergency Support to Critical Education, Health and Nutrition Services (PAUSENS) project created a basic package of health, nutrition, and reproductive health services in the district’s 13 primary health centers (Centre de Santé de Base 2), available free of charge to all pregnant women and children under 5 years [41]. This program provided equipment and medication for pharmacies and health centers, as well as training for obstetrics and neonatal care. Second, the United States Agency for International Development (USAID) funded Mikolo project provided some training and limited supervision for 150 community health workers stationed in remote villages (approximately half located inside the PIVOT-MoH catchment area) on monitoring and counseling for basic health practices in their community [42]. The main difference in healthcare provision between the PIVOT-MoH catchment area and the rest of the Ifanadiana district was the support in infrastructure building, training and staffing, removal of user fees, and additional support to guideline implementation of clinical programs provided by PIVOT.

### Data collection

#### Household surveys (IHOPE cohort)

We collaborated with Madagascar’s National Institute of Statistics (INSTAT) to create an ongoing open longitudinal cohort study of 1,600 households (the sample size required to estimate under-five mortality within a 12% margin of error) representative of the Ifanadiana district’s population—The Ifanadiana Health Outcomes and Prosperity longitudinal Evaluation (IHOPE) [34]. The households were selected through a two-stage cluster sampling scheme. Maps from the 2009 census were used to divide the district into 169 geographical clusters, after which 40 clusters from within and 40 clusters from outside the initial catchment area were chosen at random, with probabilities proportional to population size (**Fig 1**). Within each cluster, an enumeration was done to obtain a complete household listing, and 20 households were randomly selected prior to conducting the survey. A questionnaire adapted from the Demographics and Health Survey (DHS) [43] was administered in person to all men aged 15–59 and women aged 15–49 living in the enrolled households at baseline (April–May 2014). This survey was repeated two years after the initiation of the intervention (August–September 2016). Households that were unavailable or declined participation in the second survey were replaced with additional households from the original sampling lists; families and individuals that moved into original households were also included in the second survey (for details, see Miller and colleagues [34]). We collected data on household characteristics, socioeconomic status, and maternal and child health, among others. Overall, among the 1,600 households sampled in each survey, 1,522 provided data in 2014 and 1,514 in 2016 (95.1% and 94.6% response rate, respectively).

#### Health facility assessments (SARA survey)

To measure changes in service availability throughout PIVOT’s HSS intervention, we conducted facility surveys in 2014 and 2015 at the four PIVOT-support health centers in the catchment area. These were based on the WHO Service Availability and Readiness Assessment (SARA) framework [44], which was adapted to the Malagasy health system context and norms. We assessed availability of preventative and therapeutic services, health facility personnel level, supply of essential medicines, and basic functional medical equipment, among others (**S1 Appendix**). Evaluations of non-PIVOT supported health centers were not conducted at that time. Hospital-level norms for service availability and readiness were updated by the MoH during the study period, precluding a longitudinal follow-up and analysis of the district hospital from baseline values.

The IHOPE survey was approved by the Madagascar National Ethics Committee and Harvard Medical School’s IRB. Verbal consent was obtained from adults (18–59 years old) and from parents or legal guardians for their children (under 18 years), with assent from minors (15–18 years old) to participate in the study. The SARA survey was authorized by the MoH. We carried out our analysis on de-identified data. We reported this study as per the Strengthening the Reporting of Observational Studies in Epidemiology (STROBE) guidelines [45], which are available in **S1 STROBE checklist**.

### Outcomes

A prospective analysis plan for measuring changes in care seeking behavior was designed as part of the IHOPE cohort study and has been published in Miller and colleagues (2018) [34]. The current analysis of content of care was done retrospectively in 2017 to provide complementary insights around healthcare quality. We focused on three areas of healthcare: sick-child care, antenatal care, and perinatal care. To benchmark appropriate care, we used as our standard WHO’s IMCI guidelines [39], as well as their guidelines for maternal and newborn health [46,47] (**S1 Appendix**). Based on the parents’ responses, sick-child care seeking was measured as the proportion of children under five who had diarrhea (symptom of possible gastroenteritis), persistent cough with difficulty breathing (symptoms of possible acute respiratory infection), or fever (symptom of possible malaria infection) within the previous two weeks and who were brought to care for that indication either at one of MoH’s public health facilities (PHFs)—which includes the district hospital and the district’s primary health centers—or at any of the community health worker sites (CHWs) located throughout the district. The content of sick-child care was assessed by examining a variety of treatments and diagnostic procedures that ill children could receive for a particular symptom in accordance with IMCI guidelines. For each possible treatment, we measured the proportion of symptomatic children to whom it was prescribed at a PHF or CHW, as reported by parents in the IHOPE surveys.

We examined antenatal and perinatal care among women aged 15–49 who had a live birth within the previous two years. Antenatal care seeking was assessed using three indicators: the proportion of pregnant women who attended an antenatal consultation at a PHF (a) at least once in their pregnancy, (b) at least four times, and (c) at least once during their first trimester. Perinatal care seeking was measured as the proportion of women who delivered at a PHF. Content of antenatal care was assessed by examining the coverage of several screening tests pregnant women reported to have received during at least one of their antenatal consultations at a PHF. For perinatal care, we assessed the rate of recommended newborn and maternal health assessments that women reported in the IHOPE surveys following their child’s delivery at a PHF.

Based on results from the SARA surveys conducted in PIVOT-supported health centers, we examined the availability of the following services: (a) preventative services, (b) therapeutic services, (c) health promotion and administration services, and (d) complementary services (e.g., tuberculosis care, malnutrition care). We also examined health centers’ readiness to provide general healthcare services based on levels of (a) personnel (b) basic amenities (e.g., power source, water source), (c) basic equipment, and (d) essential medicines. For each indicator of service availability and readiness, we measured the available proportion of components included in the indicator, as defined by MoH/WHO standards (**S1 Appendix**).

### Data analysis

Given that the vast majority of the district’s population accesses health centers by foot, we stratified participants of the IHOPE surveys into an intervention and a non-intervention group based on geographic proximity of their household to a PIVOT-supported or nonsupported health center (**Fig 1**). Using the global positioning system (GPS) coordinates of each health center and each of the 80 geographical clusters randomly sampled (centroid of all the villages in a cluster), we identified the health center that was the shortest Euclidean distance away from a given cluster. We considered participants to be in the intervention group if they lived in households located in a cluster for which the nearest facility was a PIVOT-supported health center and in the non-intervention group otherwise.

Using the standard protocols for DHS surveys [48], all results from the IHOPE surveys were adjusted using sampling weights, which account for the unequal probability of household selection, depending on the population size of each cluster surveyed and nonresponse rates. We did not adjust for potential spatial autocorrelation through mixed effects models. We used a chi-squared statistic to assess differences between outcomes in the intervention and non-intervention group for each year, and a difference-in-differences (DiD) regression analysis to estimate the overall effect of PIVOT-MoH’s intervention over time (**S1 Appendix**). We also compared DiD estimates, unadjusted and adjusted, with household wealth. All results from the SARA surveys were averaged across the four health centers evaluated, and changes over time were reported. We analyzed all data with R software using the “survey,” “ggplot2,” “sp,” “maptools,” “rgeos,” and “foreign” packages.

## Results

The IHOPE study included 1,333 children under five in the 2014 survey and 1,345 children in the 2016 survey (31.0% and 29.7% in the intervention group, respectively). It also included 1,635 women aged 15–49 in the 2014 survey and 1,585 women in the 2016 survey (39.2% and 37.5% in the intervention group, respectively). While many demographic characteristics were similar between the two groups (**Table 1**), the household wealth index,maternal education level and literacy rate were higher in the intervention group than in the non-intervention group. The nonresponse rate was low (<2%) for all questions of care seeking behavior and care content assessed, with no significant differences between the two groups observed (**S2 Table**).

**Table 1:** Demographic characteristics of women (15–49 years old) and children (<5 years old) participants in the IHOPE cohort. Comparisons of demographic characteristics between the intervention and non-intervention groups were made separately for 2014 and 2016 surveys. Differences between groups for each survey year were calculated using a Student *t* test for continuous variables and a chi-squared test for categorical variables.

Characteristics	2014			2016		
	Non-intervention group *N* (%)	Intervention group *N* (%)	*p*-value	Non-intervention group *N* (%)	Intervention group *N* (%)	*p*-value
**Children (<5 years old)**	**920**	**413**		**945**	**400**	
**Reported sick within two weeks of survey date** *By symptom*:	474 (51.5)	183 (44.3)	0.05	313 (33.1)	98 (24.5)	0.00
Diarrhea	125 (13.6)	56 (13.6)	0.96	107 (11.3)	44 (11.0)	0.87
Fever	352 (38.3)	91 (22.0)	0.00	137 (14.5)	27 (6.8)	0.00
Cough and difficulty breathing	251 (27.3)	110 (26.6)	0.87	156 (16.5)	44 (11.0)	0.02
**Sex**			0.67			0.93
Male	465 (50.5)	203 (49.2)		484 (51.2)	204 (51.0)	
Female	455 (49.5)	210 (50.8)		461 (48.8)	196 (49.0)	
**Mean age in years**	2.5	2.6	0.39	2.8	2.6	0.27
**Mean number of siblings alive**	2.7	2.4	0.15	2.7	2.5	0.44
Household wealth index*			0.01			0.00
Within poorest 1st or 2nd wealth quintiles	516 (56.1)	144 (34.9)		525 (55.5)	141 (35.2)	
Within richest 4th or 5th wealth quintiles	236 (25.6)	191 (46.2)		212 (22.5)	189 (47.1)	
**Mean maternal age in years**	29.0	28.2	0.15	28.5	29.2	0.32
Highest maternal education level attained**			0.00			0.00
Received no formal education	360 (39.1)	89 (21.5)		360 (38.1)	99 (24.8)	
Attained primary education level	506 (55.0)	238 (57.6)		533 (56.4)	213 (53.3)	
Attained secondary education level or higher	54 (5.9)	86 (20.8)		52 (5.5)	88 (22.0)	
**Maternal literacy status**			0.00			0.00
Not literate	620 (67.4)	159 (38.5)		588 (62.2)	176 (44.0)	
Literate	299 (32.5)	254 (61.5)		356 (37.7)	221 (55.3)	
**Women (15–49 years old)**	**994**	**641**		**990**	**595**	
**Reported delivery within two years of survey date**	378 (38.0)	174 (27.1)	0.00	369 (37.3)	155 (26.1)	0.00
**Mean age in years**	28.4	28.4	0.98	28.7	29.3	0.20
**Marital status**			0.02			0.00
Not married	372 (37.4)	303 (47.3)		373 (37.7)	273 (45.9)	
Married or lives with a partner	622 (62.6)	338 (52.7)		617 (62.3)	321 (53.9)	
**Mean number of live births**	3.5	2.9	0.04	3.5	2.9	0.02
Household wealth index*			0.00			0.00
Within poorest 1st or 2nd wealth quintiles	509 (51.2)	154 (23.9)		479 (48.4)	167 (28.2)	
Within richest 4th or 5th wealth quintiles	279 (28.1)	389 (60.7)		293 (29.5)	348 (58.6)	
Highest education level attained**			0.00			0.00
Received no formal education	312 (31.4)	97 (15.1)		294 (29.7)	92 (15.5)	
Attained primary education level	597 (60.1)	318 (49.6)		588 (59.4)	301 (50.6)	
Attained secondary education level or higher	85 (8.6)	226 (35.3)		108 (10.9)	201 (33.8)	
**Literacy status**			0.00			0.00
Not literate	567 (57.0)	182 (28.4)		548 (55.4)	195 (32.8)	
Literate	423 (42.6)	459 (71.6)		440 (44.4)	398 (66.9)	

*A wealth index was calculated for each household in the Ifanadiana district based on standard DHS methods [48]. Households were categorized into quintiles (20% of Ifanadiana households in each quintile) based on their wealth index score. Households in the first and second wealth quintiles represent the poorest 40% of the population, while households in the fourth and fifth quintiles represent the richest 40% of the population.

**The education level attained was based on the highest grade level women reported to be attending or have completed at time of the survey.

Abbreviations: DHS, Demographics and Health Survey; IHOPE, The Ifanadiana Health Outcomes and Prosperity longitudinal Evaluation.

### Sick-child care

**Fig 2:**
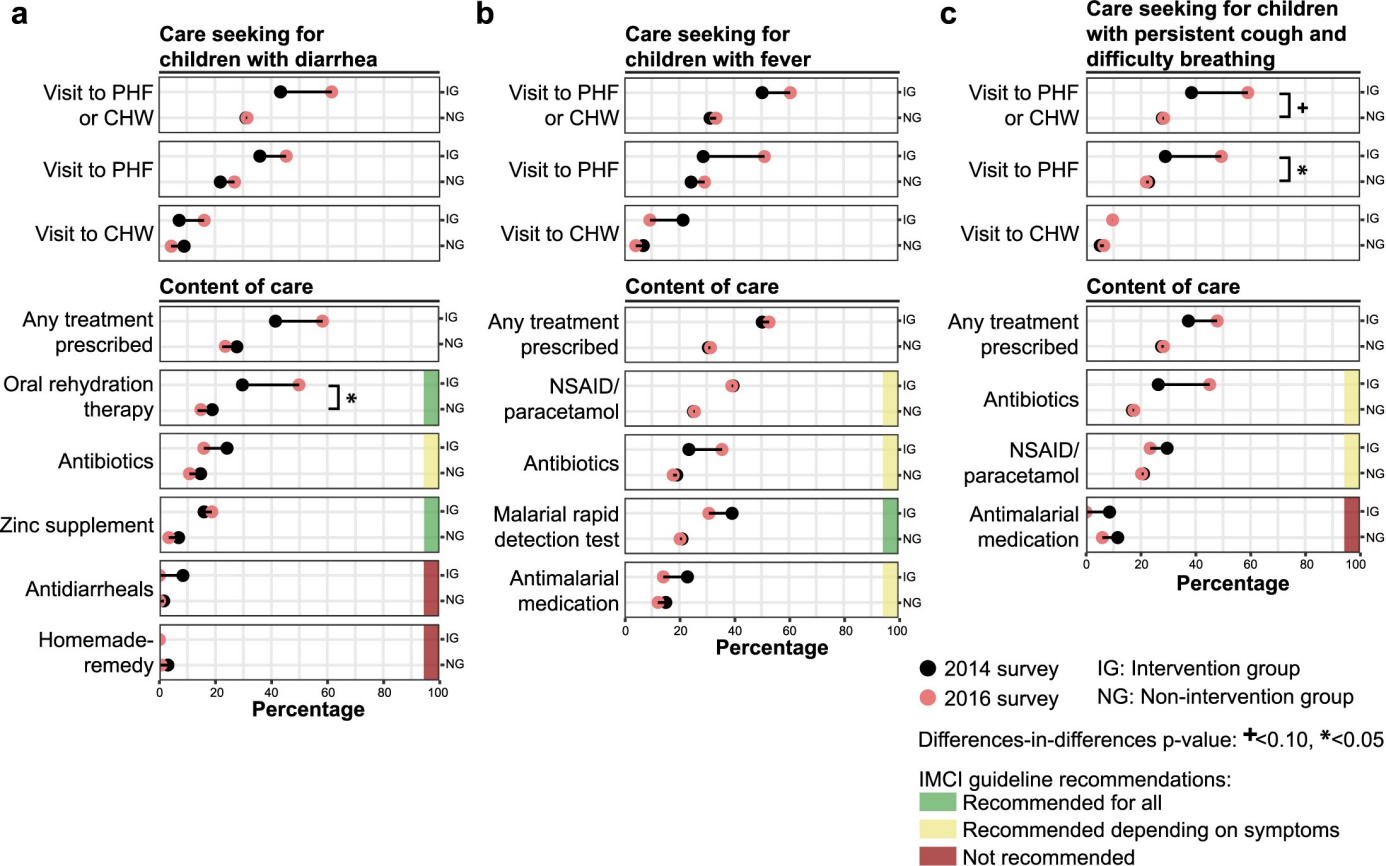
Changes in sick-child care seeking behavior and care content for children (<5 years old) over the first two years of PIVOT-MoH’s HSS intervention. Trends were calculated separately for each symptom evaluated: **(a)** diarrhea, **(b)** fever, and **(c)** persistent cough with difficulty breathing. For all outcome indicators, trends over the study period are assessed by tracing from the 2014 percentage value (black dot) to the 2016 percentage value (red dot). Recommendations for treatment are based on WHO’s IMCI guidelines (**S1 Appendix**) [39]. CHW, community health worker site; HSS, health system strengthening; IG, intervention group; IMCI, Integrated Management of Childhood Illness; MoH, Ministry of Health; NG, non-intervention group; NSAID, nonsteroidal anti-inflammatory drug; PHF, public health facility.

Over the two-year study period, the proportion of children with reported diarrhea, fever, and/or persistent coughing who sought care rose in the intervention group by 51.2% (from 41.4% in 2014 to 62.5% in 2016), compared with a 7.1% decrease (from 30.0% to 27.9%) in the non-intervention group (DiD *p*-value = 0.01) (**Table 2**). This apparent increase in care seeking behavior was predominantly attributable to increased visits to PHFs rather than to CHWs. The proportion of ill children who were prescribed at least one form of medication at a PHF or CHW appeared to increase in the intervention group by 45.2% (from 40.1% to 58.3%) and decrease in the non-intervention group by 8.9% (from 29.6% to 26.9%, DiD *p*-value = 0.02). However, trends in content of care varied widely by indicator and did not always match trends observed in care seeking behavior (**Table 2**and **Fig 2**). Among the children in the intervention group who were reported with diarrhea in 2016, 61.5% attended a PHF or CHW (+42.0% from 2014 rate) and 49.9% received oral rehydration therapy there (+68.5%), while only 18.6% received zinc supplement (+17.6%)—both of which are recommended for all children with diarrhea by the IMCI guidelines. The prescription rate for antidiarrheal medication, not recommended by IMCI guidelines, dropped from 8.3% to 0% in 2016. Among the children in the intervention group who were reported with fever in 2016, 60.5% attended a PHF or CHW (+20.4%); however, only 30.8% reported having received a malarial rapid diagnostic test (−21.6%)—a test recommended by IMCI guidelines for all children with fever in malarial endemic areas. In addition, 14.2% received antimalarial medication (−38.5%), and 35.6% received antibiotics (+51.7%), although appropriateness of these prescriptions could not be evaluated based on information reported in the IHOPE surveys. Controlling for household wealth produced similar DiD estimates and associated *p*-values for all indicators of sick-child care (**S1 Table**).

**Table 2:** Summary of responses and trends from the 2014 and 2016 IHOPE surveys on indicators of sick-child care seeking behavior and care content (children <5 years old).

Indicator		2014			2016			2014–2016 Trend		
	Study group	*N*	Percent (SE)	Difference (*p*-value)	*N*	Percent (SE)	Difference (*p*-value)	Percent relative change	Percent absolute change	DiD (*p*-value)
**Summary statistics for all children with symptoms of diarrhea, fever, and/or coughing with difficulty breathing**										
Visit to a PHF or CHW	IG	183	41.35 (5.33)	—	98	62.50 (7.42)	—	51.17	21.16	—
	NG	474	30.02 (3.35)	11.32 (0.07)^+^	313	27.88 (3.97)	34.62 (0.00)***	−7.13	−2.14	23.29 (0.01)**
Visit to a PHF	IG	183	28.98 (5.43)	—	98	49.66 (7.04)	—	71.37	20.68	—
	NG	474	22.23 (3.34)	6.75 (0.28)	313	22.10 (3.50)	27.56 (0.00)***	−0.56	−0.12	20.81 (0.00)***
Visit to a CHW	IG	183	12.85 (3.91)	—	98	12.84 (5.06)	—	−0.09	−0.01	—
	NG	474	7.98 (1.95)	4.87 (0.23)	313	5.78 (2.52)	7.06 (0.17)	−27.61	−2.20	2.19 (0.74)
Any treatment prescribed at a PHF or CHW	IG	183	40.14 (5.53)	—	98	58.26 (7.73)	—	45.16	18.12	—
	NG	474	29.56 (3.31)	10.58 (0.10)^+^	313	26.93 (3.95)	31.33 (0.00)***	−8.89	−2.63	20.75 (0.02)*
**Care seeking for children with diarrhea**										
Visit to a PHF or CHW	IG	56	43.28 (9.88)	—	44	61.47 (8.41)	—	42.03	18.19	—
	NG	125	31.02 (5.90)	12.26 (0.28)	107	31.45 (5.30)	30.02 (0.00)***	1.37	0.43	17.76 (0.16)
Visit to a PHF	IG	56	35.96 (10.01)	—	44	45.29 (10.78)	—	25.95	9.33	—
	NG	125	21.95 (5.27)	14.01 (0.19)	107	26.97 (5.30)	18.32 (0.12)	22.87	5.02	4.31 (0.74)
Visit to a CHW	IG	56	7.32 (4.97)	—	44	16.18 (9.15)	—	120.98	8.86	—
	NG	125	9.07 (3.37)	-1.75 (0.78)	107	4.48 (1.86)	11.70 (0.06)^+^	−50.63	−4.59	13.45 (0.21)
**Content of care for children with diarrhea**										
Any treatment prescribed at a PHF or CHW	IG	56	41.50 (10.04)	—	44	58.30 (8.80)	—	40.48	16.80	—
	NG	125	27.69 (6.23)	13.80 (0.23)	107	22.42 (4.75)	35.88 (0.00)***	−19.05	−5.28	22.07 (0.13)
Oral rehydration therapy prescribed at a PHF or CHW	IG	56	29.64 (6.81)	—	44	49.93 (10.52)	—	68.48	20.30	—
	NG	125	17.78 (5.47)	11.86 (0.18)	107	13.65 (3.37)	36.28 (0.00)***	−23.22	−4.13	24.43 (0.05)*
Antibiotics prescribed at a PHF or CHW	IG	56	24.19 (7.08)	—	44	15.88 (7.09)	—	−34.34	−8.31	—
	NG	125	14.71 (3.89)	9.48 (0.21)	107	10.71 (3.08)	5.17 (0.46)	−27.18	−4.00	−4.31 (0.67)
Zinc supplements prescribed at a PHF or CHW	IG	56	15.81 (4.66)	—	44	18.59 (11.47)	—	17.61	2.78	—
	NG	125	6.78 (2.80)	9.03 (0.09)^+^	107	3.19 (1.79)	15.40 (0.03)*	−52.97	−3.59	6.38 (0.65)
Antidiarrheal medication prescribed at a PHF or CHW	IG	56	8.32 (6.66)	—	44	0.00 (0.00)	—	−100.00	−8.32	—
	NG	125	1.52 (1.08)	6.80 (0.08)^+^	107	0.00 (0.00)	—	−100.00	−1.52	−6.80 (0.32)
Homemade remedy prescribed at a PHF or CHW	IG	56	0.00 (0.00)	—	44	0.00 (0.00)	—	0.00	0.00	—
	NG	125	2.97 (2.09)	−2.97 (0.34)	107	0.53 (0.53)	−0.53 (0.54)	−82.33	−2.45	2.45 (0.26)
**Care seeking for children with persistent cough and difficulty breathing**										
Visit to a PHF or CHW	IG	110	38.46 (5.32)	—	44	58.97 (11.24)	—	53.31	20.50	—
	NG	251	27.85 (4.05)	10.61 (0.11)	156	28.29 (4.54)	30.67 (0.01)**	1.58	0.44	20.06 (0.08)^+^
Visit to a PHF	IG	110	28.83 (5.63)	—	44	49.28 (10.48)	—	70.97	20.46	—
	NG	251	22.77 (3.67)	6.06 (0.36)	156	21.90 (3.96)	27.38 (0.01)**	−3.80	−0.87	21.32 (0.03)*
Visit to a CHW	IG	110	9.64 (3.15)	—	44	9.68 (5.21)	—	0.48	0.05	—
	NG	251	5.08 (1.86)	4.55 (0.19)	156	6.39 (3.13)	3.29 (0.57)	25.71	1.31	−1.26 (0.84)
**Content of care for children with persistent cough and difficulty breathing**										
Any treatment prescribed at a PHF or CHW	IG	110	37.35 (5.81)	—	44	47.85 (10.38)	—	28.10	10.50	—
	NG	251	27.54 (4.05)	9.81 (0.16)	156	28.29 (4.54)	19.56 (0.07)^+^	2.73	0.75	9.74 (0.40)
Antibiotics prescribed at a PHF or CHW	IG	110	26.32 (4.83)	—	44	45.12 (10.43)	—	71.43	18.80	—
	NG	251	16.95 (3.65)	9.37 (0.12)	156	17.47 (3.48)	27.65 (0.00)***	3.08	0.52	18.28 (0.13)
NSAIDs/paracetamol prescribed at a PHF or CHW	IG	110	29.59 (4.69)	—	44	23.40 (6.85)	—	−20.92	−6.19	—
	NG	251	21.03 (3.81)	8.56 (0.16)	156	20.30 (4.02)	3.10 (0.69)	−3.48	−0.73	−5.46 (0.48)
Antimalarial medication prescribed at a PHF or CHW	IG	110	8.61 (2.89)	—	44	0.00 (0.00)	—	−100.00	−8.61	—
	NG	251	11.56 (2.43)	−2.95 (0.45)	156	5.90 (2.53)	−5.90 (0.18)	−48.98	−5.66	−2.95 (0.46)
**Care seeking for children with fever**										
Visit to a PHF or CHW	IG	91	50.26 (6.38)	—	27	60.52 (12.36)	—	20.41	10.26	—
	NG	352	31.25 (3.87)	19.00 (0.01)**	137	33.48 (6.00)	27.03 (0.05)*	7.13	2.23	8.03 (0.57)
Visit to a PHF	IG	91	28.83 (7.31)	—	27	51.15 (12.25)	—	77.44	22.32	—
	NG	352	24.37 (4.16)	4.46 (0.59)	137	29.29 (5.74)	21.86 (0.10)^+^	20.18	4.92	17.41 (0.25)
Visit to a CHW	IG	91	21.43 (5.97)	—	27	9.36 (5.83)	—	−56.30	−12.07	—
	NG	352	6.88 (1.77)	14.55 (0.00)***	137	4.20 (2.17)	5.17 (0.32)	−39.04	−2.69	−9.38 (0.30)
**Content of care for children with fever**										
Any treatment prescribed at a PHF or CHW	IG	91	50.26 (6.38)	—	27	52.76 (12.59)	—	4.99	2.51	—
	NG	352	30.63 (3.79)	19.63 (0.01)**	137	31.37 (5.79)	21.39 (0.12)	2.43	0.74	1.76 (0.90)
NSAIDs/paracetamol prescribed at a PHF or CHW	IG	91	39.63 (4.91)	—	27	39.16 (10.50)	—	−1.18	−0.47	—
	NG	352	25.20 (3.83)	14.43 (0.02)*	137	25.60 (5.27)	13.57 (0.23)	1.57	0.40	−0.87 (0.95)
Antibiotics prescribed at a PHF or CHW	IG	91	23.49 (4.45)	—	27	35.63 (11.75)	—	51.67	12.14	—
	NG	352	19.13 (3.10)	4.36 (0.42)	137	17.78 (5.12)	17.85 (0.13)	−7.08	−1.35	13.49 (0.31)
Malarial rapid diagnostic test administered at a PHF or CHW	IG	91	39.26 (7.50)	—	27	30.78 (9.97)	—	−21.60	−8.48	—
	NG	352	20.98 (4.00)	18.28 (0.03)*	137	20.28 (4.66)	10.50 (0.31)	−3.30	−0.69	−7.79 (0.53)
Antimalarial medication prescribed at a PHF or CHW	IG	91	22.99 (5.20)	—	27	14.15 (7.10)	—	−38.45	−8.84	—
	NG	352	15.10 (2.60)	7.89 (0.15)	137	12.24 (4.15)	1.91 (0.81)	−18.92	−2.86	−5.98 (0.50)

^+^*p*-value < 0.10.

*p-value < 0.05.

**p-value < 0.01.

***p-value < 0.001.

Abbreviations: CHW, community health worker site; DiD, difference-in-differences; IG, intervention group; IHOPE, The Ifanadiana Health Outcomes and Prosperity longitudinal Evaluation; NG, non-intervention group; NSAID, nonsteroidal anti-inflammatory drug; PHF, public health facility.

### Antenatal and perinatal care

**Fig 3:**
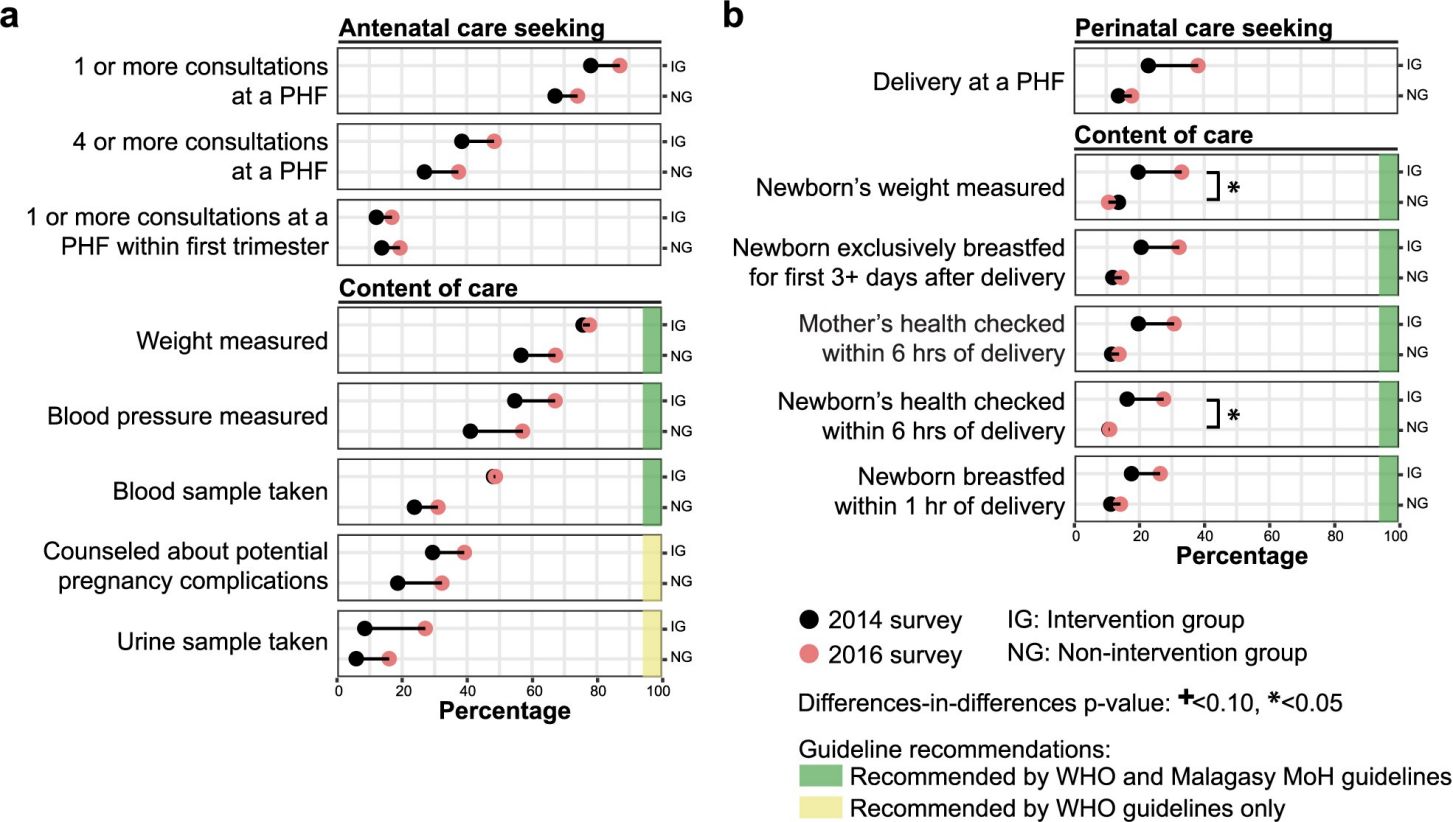
Changes in maternal and newborn care seeking behavior and care content over the first two years of PIVOT-MoH’s HSS intervention. Trends were calculated for **(a)** antenatal care and **(b)** perinatal care among women (15–49 years old) who had a delivery within two years of the 2014 and 2016 IHOPE surveys. For all outcome indicators, trends over the study period are assessed by tracing from the 2014 percentage value (black dot) to the 2016 percentage value (red dot). Recommendations for care are based on WHO guidelines (**S1 Appendix**) [46,47]. HSS, health system strengthening; IG, intervention group; IHOPE, The Ifanadiana Health Outcomes and Prosperity longitudinal Evaluation; MoH, Ministry of Health; NG, non-intervention group; PHF, public health facility; WHO, World Health Organization.

Between 2014 and 2016, antenatal care seeking behavior appeared to increase moderately for pregnant women in the intervention group (**Table 3**and **Fig 3**). In the 2016 IHOPE survey, 87.2% of these women attended an antenatal consultation at a PHF at least once during their pregnancy (+11.4% increase from 2014 survey rate); however, only 48.5% attended four or more consultations throughout their pregnancy, and 17.0% attended a consultation within the first trimester of their pregnancy. Results are detailed in **S1 Appendix**. With regards to content of antenatal care, there were variable increases over the study period in the rates of standard screening tests and measurements, and many indicators remained low in 2016 despite the apparent increase in care seeking behavior (**Table 3**and **Fig 3**). While 77.8% of pregnant women in the intervention group reported in the 2016 survey to have attended at least one consultation at a PHF and had their weight measured (+2.8%), 67.1% had their blood pressure measured (+22.8%), 48.7% had a blood test (+1.1%), and 27.1% had a urine test (+219.4%) during at least one of their consultations—all measurements, except urine tests, recommended by both international and national antenatal care guidelines. Moreover, only 39.1% of pregnant women reported receiving counseling on how to address possible pregnancy complications (+33.4%). No significant differences in trends for care seeking behavior or content of antenatal care were observed between the intervention and non-intervention groups, although the intervention group appeared to have consistently higher rates for all indicators.

In contrast to antenatal care, perinatal care seeking behavior appeared to increase substantially for pregnant women in the intervention group over the study period, and trends in the content of perinatal care more closely paralleled those observed for care seeking (**Table 3**and **Fig 3**). The percentage of pregnant women in the intervention group who delivered a child at a PHF appeared to increase by 66.2% over the study period (23.1% to 38.3%). Similarly, all content of perinatal care indicators examined appeared to increase by 50%–70% over the study period, and most of these indicators had 2016 rates above 30%, similar to the 2016 rate of PHF-based delivery. In comparison, indicators of care seeking behavior and content of perinatal care for the non-intervention group appeared to minimally change over the study period and remained low in 2016 (2016 rates <18% for all indicators, with <30% increases from 2014 rates).

**Table 3:** Summary of responses and trends from the 2014 and 2016 IHOPE surveys on indicators of antenatal and perinatal care seeking behavior and care content for women (15–49 years old) and newborns.

Indicator		2014			2016			2014–2016 Trend		
	Study group	*N*	Percent (SE)	Difference (*p*-value)	*N*	Percent (SE)	Difference (*p*-value)	Percent relative change	Percent absolute change	DiD (*p*-value)
**Antenatal care seeking for pregnant women**										
1 or more consultations at a PHF	IG	174	78.25 (5.48)	—	155	87.20 (3.84)	—	11.44	8.95	—
	NG	378	67.27 (4.40)	10.98 (0.14)	369	74.21 (4.74)	12.99 (0.04)*	10.33	6.95	2.01 (0.75)
4 or more consultations at a PHF	IG	174	38.52 (5.68)	—	155	48.45 (5.86)	—	25.78	9.93	—
	NG	378	26.98 (3.50)	11.54 (0.08)^+^	369	37.45 (5.24)	11.00 (0.17)	38.79	10.47	−0.54 (0.95)
1 or more consultations at a PHF within the first trimester	IG	174	12.16 (2.67)	—	155	16.95 (3.93)	—	39.48	4.80	—
	NG	378	13.89 (2.39)	−1.73 (0.63)	369	19.45 (3.21)	−2.50 (0.63)	40.05	5.56	−0.76 (0.90)
**Content of antenatal care for pregnant women**										
Weight measured at a PHF	IG	174	75.69 (5.95)	—	155	77.80 (3.81)	—	2.79	2.11	—
	NG	378	56.61 (4.70)	19.08 (0.02)*	369	67.22 (5.55)	10.58 (0.11)	18.74	10.61	−8.50 (0.18)
Blood pressure measured at a PHF	IG	174	54.67 (5.86)	—	155	67.12 (4.21)	—	22.78	12.45	—
	NG	378	41.03 (3.72)	13.64 (0.05)*	369	57.15 (5.57)	9.97 (0.15)	39.28	16.12	−3.67 (0.66)
Blood sample taken at a PHF	IG	174	48.18 (5.83)	—	155	48.69 (5.01)	—	1.06	0.51	—
	NG	378	23.72 (3.85)	24.46 (0.00)***	369	31.00 (4.40)	17.69 (0.01)**	30.71	7.28	−6.77 (0.29)
Counseled about potential pre- gnancy complications at a PHF	IG	174	29.33 (4.90)	—	155	39.13 (5.15)	—	33.41	9.80	—
	NG	378	18.64 (2.76)	10.68 (0.05)*	369	32.25 (4.48)	6.88 (0.32)	72.98	13.61	−3.81 (0.58)
Urine sample taken at a PHF	IG	174	8.49 (2.74)	—	155	27.11 (4.57)	—	219.4	18.63	—
	NG	378	5.79 (1.47)	2.70 (0.35)	369	16.00 (2.31)	11.12 (0.02)*	176.52	10.21	8.41 (0.19)
**Perinatal care seeking for pregnant women and newborns**										
Delivery at a PHF	IG	174	23.06 (4.40)	—	155	38.31 (7.63)	—	66.15	15.25	—
	NG	378	13.87 (4.18)	9.19 (0.15)	369	17.87 (3.61)	20.44 (0.01)**	28.87	4.00	11.25 (0.13)
**Content of perinatal care for pregnant women and newborns**										
Newborn’s weight measured at a PHF	IG	174	19.62 (4.34)	—	155	33.00 (6.88)	—	68.17	13.38	—
	NG	378	13.53 (4.13)	6.09 (0.32)	369	10.44 (2.68)	22.56 (0.00)***	-22.88	−3.10	16.47 (0.02)*
Newborn exclusively breastfed for first 3 or more days after delivery at a PHF	IG	174	20.52 (4.16)	—	155	32.25 (6.51)	—	57.14	11.73	—
	NG	378	11.82 (3.46)	8.70 (0.12)	369	14.52 (3.03)	17.73 (0.01)**	22.83	2.70	9.03 (0.16)
Mother’s health checked at a PHF within 6 hours of delivery	IG	174	19.71 (3.69)	—	155	30.70 (6.21)	—	55.78	10.99	—
	NG	378	11.41 (3.50)	8.30 (0.12)	369	13.67 (3.30)	17.03 (0.01)**	19.76	2.26	8.74 (0.12)
Newborn’s health checked at a PHF within 6 hours of delivery	IG	174	16.25 (3.40)	—	155	27.43 (5.93)	—	68.82	11.18	—
	NG	378	10.58 (3.05)	5.66 (0.23)	369	10.80 (2.34)	16.63 (0.00)***	2.03	0.21	10.97 (0.04)*
Newborn breastfed within 1 hour of delivery at a PHF	IG	174	17.56 (3.79)	—	155	26.41 (6.66)	—	50.39	8.85	—
	NG	378	11.16 (4.13)	6.40 (0.28)	369	14.15 (3.20)	12.26 (0.07)^+^	26.78	2.99	5.86 (0.43)

^+^*p*-value < 0.10.

*p-value < 0.05.

**p-value < 0.01.

***p-value < 0.001.

Abbreviations: CHW, community health worker site; DiD, difference-in-differences; IG, intervention group; IHOPE, The Ifanadiana Health Outcomes and Prosperity longitudinal Evaluation; NG, non-intervention group; PHF, public health facility.

### Service availability and readiness in PIVOT-MoH catchment

Over the first year of the HSS intervention, there was an apparent increase in the mean availability of services in PIVOT-support health centers, across all categories measured in the SARA surveys **(Table 4)**: in 2015, the average health center offered 100.0% of the minimum required preventative services (+14.3% increase from 2014 levels), 100.0% of health promotion and administration services (+9.1%), 90.0% of therapeutic services (+20.0%), and 41.7% of complementary services (+25.0%). Moreover, there was an apparent increase in most categories of general service readiness: in 2015, the average health center had 83.3% of the basic functional medical equipment components (+66.7%) and 65.0% of the basic health facility amenities (+85.7%). However, only 58.3% of essential medicines were in provision (−23.9%), and the minimum level of health facility personnel required by MoH standards remained at 75.0% both years. Comparisons with service availability and readiness levels in health centers outside the PIVOT-MoH intervention catchment could not be performed because those health centers were not surveyed at the time.

**Table 4:** Summary of operational capacities indicators from the 2014 and 2015 SARA surveys. The surveys were conducted at all PIVOT-supported health centers located inside the PIVOT-MoH catchment area. Percentages reported are the available proportion of components in each indicator, averaged across the four health centers assessed.

Indicator	Components of indicator	2014 Percent (SD)	2015 Percent (SD)	Percent relative change	Percent absolute change
**Specific service availability**					
Preventative services	antenatal care, postnatal care, family planning, childhood immunization	87.50 (14.43)	100.00 (0.00)	14.29	12.50
Therapeutic services	outpatient care, inpatient care, obstetric care, hospitalization capabilities, treatment capabilities	75.00 (19.15)	90.00 (20.00)	20.00	15.00
Health promotion and administration services	health promotion, community outreach, health management information system	91.67 (16.67)	100.00 (0.00)	9.09	8.33
Complementary services	malnutrition care, tuberculosis care, IMCI guidelines implementation	33.33 (0.00)	41.67 (16.67)	25.00	8.33
*Overall score*		73.33 (5.44)	85.00 (3.33)	15.91	11.67
**General service readiness**					
Personnel level	1 physician, 1 nurse, 1 midwife, 1 guard, 1 pharmacist	75.00 (25.17)	75.00 (25.17)	0.00	0.00
Basic amenities	power, improved water source, adequate sanitation facilities, communication equipment, sufficient work space	35.00 (25.17)	65.00 (25.17)	85.71	30.00
Basic equipment	adult scale, child scale, thermometer, stethoscope, blood pressure apparatus, examination table	50.00 (0.00)	83.33 (13.61)	66.67	33.33
Essential medicines	aminophylline, amoxicillin, benzylpenicillin, captopril, chlorpheniramine, co-trimoxazole, iron-folic acid, gentamicin, hydrochlorothiazide, ibuprofen, metoclopramide, metronidazole, paracetamol, phenobarbital, oral rehydration therapy/zinc packets	76.67 (8.61)	58.33 (18.98)	−23.91	−18.33
*Overall score*		64.52 (8.33)	69.00 (3.83)	6.95	4.48

Abbreviations: IMCI, Integrated Management of Childhood Illness; MoH, Ministry of Health; SARA, Service Availability and Readiness Assessment.

## Discussion

Of the 15.6 million avertable deaths that occurred in 2016 in low- and middle-income countries, an estimated 3.6 million were attributable to non-utilization of healthcare services, while 5.0 million were attributable to receipt of low-quality care [49]. In this study, we used an open district-representative longitudinal cohort to assess the content of care delivered to the population in the Ifanadiana district as a proxy for estimating the impact of an integrated HSS initiative on healthcare quality. The results revealed that over the two-year study period, care seeking behavior appeared to have substantially increased in the intervention group compared with the non-intervention group for sick-child care (DiD = 23.3%, *p*-value = 0.01) and perinatal care (DiD = 11.3%, *p*-value = 0.13), with a more marginal difference observed between the two groups for antenatal care (DiD = 2.0%, *p*-value = 0.75).

Despite general improvements in care seeking behavior, there was high variability in the trends observed for indicators of healthcare content in the intervention group. Among children ill with diarrhea, we observed a large increase in the prescription rate of oral rehydration therapy, matching the apparent increase in care seeking behavior, but a negligible increase for zinc supplementation. We found decreasing rates of malarial rapid diagnostic test administration and antimalarial medication prescriptions over the study period, compared with an increasing percentage of children ill with fever who attended a PHF. These discrepancies may be partially explained as artifacts of when and how the data were collected, including sample size limitations, recall bias, and imperfect knowledge from mothers on the care their children received. Indeed, sample size for children ill with fever was low in the intervention group, given that this group had lower prevalence of fever than the non-intervention group both years. Moreover, the 2016 IHOPE survey was conducted between August and September, which was four months later in the year than the 2014 survey and when the incidence rates of malaria and other infectious diseases are lower due to a drier climate.

We also observed a parallel increase in the care seeking and content of perinatal care during the study period, but variable changes in the content of antenatal care. By the second wave of the IHOPE survey in 2016, the PIVOT-MoH partnership had been primarily focused on improving facility readiness and had not yet implemented specific clinical programs for maternal and reproductive health. In addition, while many health facility readiness programs started in mid-2014, the support to the community health worker program was only initiated in November 2015. This could explain why changes in sick-child care seeking at CHWs were marginal. Despite these limitations, the specific gaps observed between trends in care content and care seeking behavior highlight the fact that measures of care seeking are necessary but not sufficient to adequately evaluate the impact of HSS programs on delivering effective healthcare.

The high variability we found in the trends of care content across outputs examined at the local level is consistent with recent evidence observed at larger scales in studies comparing primary healthcare across several low-income countries [23,50,51]. Such analyses show that even among some populations with high coverage rates, large gaps in the content of care persist, resulting in inadequate management of patients [24,25,52,53]. Moreover, studies have found only weak correlations between care performance in low-income countries, facility infrastructure, and national wealth [26,50,54,55], suggesting that a focus on improving care content (i.e., by emphasizing consistent clinical assessment, adherence to protocols, respectful patient-provider interactions [56]) could achieve substantial improvements in care quality in these settings.

In the context of localized HSS impact studies, there has been limited use of content of care measures to explore care quality at the population level [7]. Content of care has most commonly been assessed by evaluating the practices of healthcare providers directly at health centers [57–59]. This method enables more detailed examination of services than is possible from population surveys (e.g., correct diagnosis, appropriate medicine dosage), but can introduce participation bias if providers and patients know they are being evaluated [16]. Moreover, using direct observational methods alone only accounts for people who reach the health facilities and precludes complementary measures of accessibility.

We also noted improvements in most measurements of service availability and readiness in the PIVOT-supported health centers. Within the first year of the intervention, all four PIVOT-supported health centers in the catchment area appeared to function close to full operational capacity, offering almost all preventative and therapeutic services, as well as becoming well supplied with basic health facility amenities and functional medical equipment. However, supply chain issues were pervasive and the provision of essential medicines remained at low levels in 2015 and decreased from 2014 levels. Medications for all health centers in the Ifanadiana district are ordered from a single regional distribution system; shortages of essential medicines in PIVOT-supported health centers are indicative of challenges in the supply chain keeping up with the increased demand, revealing a critical bottleneck in the healthcare delivery system that required improvement. SARA surveys conducted throughout Madagascar in 2014 indicate that about half of health centers lack stocks of zinc and oral rehydration therapy [60]. Moreover, similar data from multiple low-income countries reveal that the rate of essential medicines’ availability is consistently low compared with other indicators measured in general service readiness [61].

Previous studies examining service readiness and availability in low- and middle-income countries have most commonly employed WHO’s SARA tool or DHS and USAID’s Service Provision Assessment (SPA) tool. Recently, researchers have also used the “clinical cascade” model to measure service-specific readiness, which organizes results hierarchically based on the order of variables necessary to sequentially identify, treat, and monitor or modify a particular health condition [62–64]. This methodology is clinically focused and less applicable to measures of general service readiness, which was the aim of this current study.

There were several limitations to this study. While we surveyed close to 1,600 households in each IHOPE survey, and over 400 children were reported to be ill within two weeks of each survey date, those children were separated into three groups based on symptoms in order to examine appropriate care, producing smaller sample sizes for each group. As a result, some estimates had large standard errors, which may explain, e.g., the minor apparent decreases observed in the non-intervention group despite the complementary health programs in place. In addition, data were based on self-reported answers from mothers, which could lead to lower estimates of illness or treatment if mothers did not recall the symptoms or treatments provided. Questions on content of medical care for childhood illnesses and maternal health were limited, as the IHOPE survey was largely based on the Malagasy DHS, making it challenging to assess the appropriateness of certain drug prescriptions for individual patients. Complementing population-level surveys with direct observational surveys in health facilities, as discussed above, could help address questions of care appropriateness and issues of recall. Additionally, apparent increases in essential medications and basic equipment availability observed in the SARA survey could in part be attributable to either of the programs (PIVOT or PAUSENS) that strengthened health facilities evaluated. The lack of a control group in the SARA survey (only PIVOT-supported health centers were assessed) prevents us from isolating the effect of each program.

Regarding the study design, the IHOPE open longitudinal cohort confers some key advantages over multiple cross-sectional studies. In particular, a longitudinal study is a powerful way of controlling for the confounding effects of population and demographic changes through migration (i.e., it measures the effects of the intervention on the families and communities that were present at the beginning of the intervention). However, the cohort population can become less representative of the district population over time, and substantial loss to follow-up (which was not a substantial problem in the first two years of this study) can eventually limit analysis. For this reason, the IHOPE cohort replaces households unable or unwilling to participate in data collection with supplemental households from the original enumeration list (within the same cluster), which allows for some evolving representativeness of the population estimates. We also note that the intervention catchment area was not randomized across the district—it was a natural experiment in which study design followed the intervention, which was driven by practical considerations to strengthen the health system in alignment with the government. Although health seeking behaviors at baseline were similar between the two areas, some socioeconomic indicators were better within the intervention catchment than for the rest of the district, which could impact the results observed and limit our capacity to ensure that the parallel trends assumption was fulfilled. Nonetheless, we found that DiD estimates for all indicators were extremely similar when controlling for household wealth. Lastly, because clusters were based on geographic proximity, there is a risk for misclassification if people preferred to travel longer distances to attend an improved health center, which could underestimate the effect of the intervention.

### Conclusion

Examining the rates of healthcare outputs at the population level, in addition to care seeking behavior, presents a more comprehensive picture of a program’s impact on healthcare delivery. By controlling for baseline differences between the intervention group and the non-intervention group, we were able to identify areas of care that appeared to have significantly improved as a direct result of PIVOT-MoH’s initiatives and those that still need improvement. As the PIVOT-MoH partnership continues to develop its clinical programs, particularly in maternal and reproductive health, we expect to observe a better match between the trends in healthcare seeking behavior and content of recommended care. This will be assessed in subsequent surveys of IHOPE’s ongoing longitudinal cohort study. In addition, an expansion of health facility assessments is expected to provide complementary information on health system readiness across the Ifanadiana district. Together, this will help build an evidence base for the potential impact of integrated health systems strengthening interventions on the quality of care delivered in low-resource settings.

## Supporting information

S1 Appendix The file contains the following: A table of 10 essential components of high-quality health systems; a graphical representation of the DiD statistical calculation; a figure of initiation and frequency of antenatal care attendance in the IHOPE cohort; additional information on the IHOPE cohort; and additional information on the SARA survey. DiD, difference-in-differences; IHOPE, The Ifanadiana Health Outcomes and Prosperity longitudinal Evaluation; SARA, Service Availability and Readiness Assessment.
(DOCX)

S1 TIDIER Checklist Summary of the HSS intervention carried out by PIVOT in Ifanadiana District between 2014 and 2016, based upon the TIDieR checklist. HSS, health system strengthening; TIDieR, Template for Intervention Description and Replication.
(DOCX)

S1 STROBE Checklist The STROBE statement is a checklist of 22 items considered essential for good reporting of cohort studies. STROBE, Strengthening the Reporting of Observational Studies in Epidemiology.
(DOCX)

S1 Table DiD estimates adjusted for household wealth. Table of DiD estimates and associated *p*-values for all indicators of sick-child, antenatal, and perinatal care assessed, comparing unadjusted and adjusted values after controlling for household wealth. DiD, difference-in-differences.
(XLSX)

S2 Table Information on nonresponse rates in IHOPE survey. Table with rates of missing data for all indicators of sick-child, antenatal, and perinatal care assessed. IHOPE, The Ifanadiana Health Outcomes and Prosperity longitudinal Evaluation.
(XLSX)

## Abbreviations

CHW: community health worker site
DHS: Demographics and Health Survey
DiD: difference-in-differences
GPS: global positioning system
HSS: health system strengthening
IHOPE: The Ifanadiana Health Outcomes and Prosperity longitudinal Evaluation
IMCI: Integrated Management of Childhood Illness
INSTAT: National Institute of Statistics
MoH: Ministry of Health
NSAID: nonsteroidal anti-inflammatory drug
PAUSENS: Emergency Support to Critical Education, Health and Nutrition Services
PHF: public health facility; SARA, Service Availability and Readiness Assessment
SDG: Sustainable Development Goal
SPA: Service Provision Assessment
STROBE: Strengthening the Reporting of Observational Studies in Epidemiology
TIDieR: Template for Intervention Description and Replication
UHC: universal health coverage
USAID: United States Agency for International Development
WHO: World Health Organization
