# Supplementary material for: Assessing trends in the content of maternal and child care following a health system strengthening initiative in rural Madagascar: A longitudinal cohort study
Source: PLoS Med. 2019 Aug 20;16(8):e1002869. doi: 10.1371/journal.pmed.1002869 (PMC6701767; doi:10.1371/journal.pmed.1002869)
Supplement: S1 Appendix — DiD, difference-in-differences; IHOPE, The Ifanadiana Health Outcomes and Prosperity longitudinal Evaluation; SARA, Service Availability and Readiness Assessment. (DOCX) [file pmed.1002869.s001.docx]

**S1 Appendix**

**Assessing trends in the content of maternal and child care following a health system strengthening initiative in rural Madagascar: a longitudinal cohort study**

Camille Ezran^1*^, Matthew H. Bonds^2,3^, Ann C. Miller^3^, Laura F. Cordier^2^, Justin Haruna^2^, David Mwanawabenea^2^, Marius Randriamanambintsoa^4^, Hery-Tiana R. Razanadrakato^4^, Mohammed Ali Ouenzar^2^, Bénédicte R. Razafinjato^2^, Megan Murray^3^, Andres Garchitorena^2,5*^

1. Department of Health Research and Policy, Stanford University School of Medicine, Stanford, California, USA
2. PIVOT, Ranomafana, Madagascar
3. Department of Global Health and Social Medicine, Harvard Medical School, Blavatnik Institute, Boston, Massachusetts, USA
4. Direction de la Démographie et des Statistiques Sociales, Institut National de la Statistique, Antananarivo, Madagascar
5. MIVEGEC, Univ Montpellier, CNRS, IRD, Montpellier, France

*E-mail: camillee@stanford.edu and andres.garchitorena@gmail.com

**Appendix 1. 10 essential components of high-quality health systems.** A conceptual framework to evaluate health systems, established by The Lancet Global Health Commission on High Quality Health Systems in the SDG Era established [1]. Column on the right indicates which components of the framework were assessed in this evaluation of the PIVOT-MoH HSS intervention in the Ifanadiana district of Madagascar.

| **Components of high-quality health systems** | | **Assessed in**  **this study?** |
| --- | --- | --- |
| ***Quality of impacts*** | | |
| **Better health** | Reduced mortality and morbidity, positive wellbeing and function, avoidance of serious health-related suffering | **🗶** |
| **Confidence in system** | Trust in health works and appropriate care uptake | **🗶** |
| **Economic benefit** | Ability to work or attend school, economic growth, reduction in health system waste, financial risk protection | **🗶** |
| ***Processes of care*** | | |
| **Competent care and systems** | Systematic assessment, appropriate treatment, counselling, referral, safety, prevention, continuity and integration, timely action | **✓**  **(IHOPE survey)** |
| **Positive user experience** | Dignity of patient, privacy, non-discrimination, autonomy, clear communication, short wait times, patient voice and values, ease of use | **🗶** |
| ***Foundations*** | | |
| **Population** | Individuals, families, and communities as partners and providers in providing healthcare and improving health outcomes | **🗶** |
| **Governance** | Strong leadership and financing, political commitment, policies for the public and private sector, institutions for accountability and evaluation | **🗶** |
| **Platforms** | Appropriate number and distribution of facilities, service mix, geographic access, functioning connections between levels of care | **✓**  **(SARA survey)** |
| **Workforce** | Adequate numbers of healthcare providers and managers, training in ethics, professionalism, supportive environment | **✓**  **(SARA survey)** |
| **Tools** | Availability of equipment and medicines, information systems, culture of quality, use of data, supervision, feedback | **✓**  **(SARA survey)** |

Appendix 2. Graphical representation of the difference-in-differences (DiD) statistical calculation. Subtracting the change observed in the non-intervention group over the two-year study period (B-A) from the change observed in the intervention group over the same time period (D-C) controls for extraneous contributors to the outcome variable that are not attributable to the PIVOT-MoH's intervention. DiD is equivalent to a regression model that tests for the interaction of time and strata group.


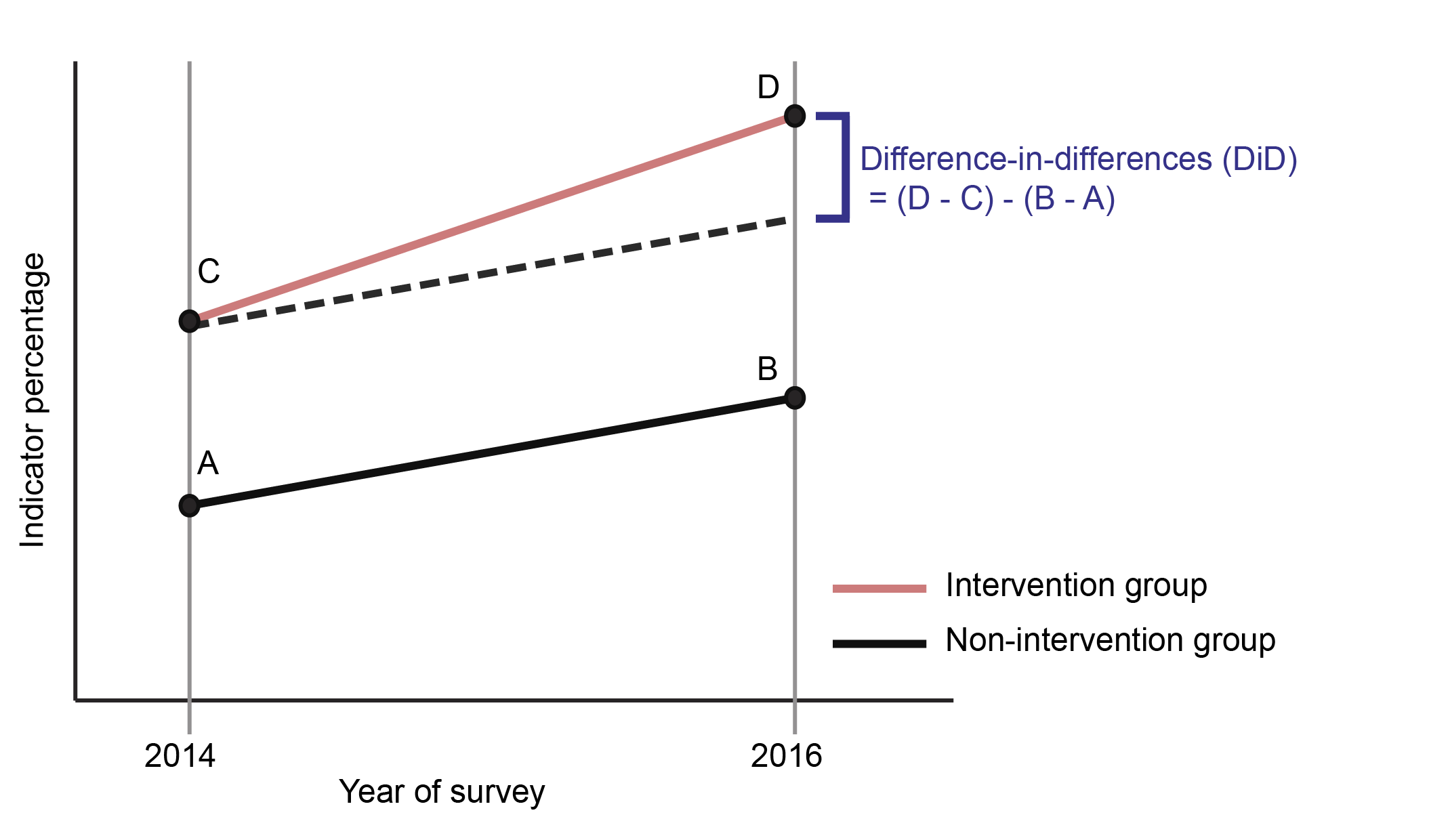


Appendix 3. Initiation and frequency of antenatal care attendance for pregnant women (15-49 years old) in the IHOPE cohort over the first two years of PIVOT-MoH’s HSS intervention. a) Distribution of number of antenatal consultations attended at a PHF among pregnant women who attended at least one consultation, separated by intervention group (red curves) and non-intervention group (black curves). In parentheses, the mean number of consultations attended throughout pregnancy. b) Distribution of pregnancy month when women reported to have attended their first antenatal consultation at a PHF. In parentheses, the mean pregnancy month of first consultation attended.


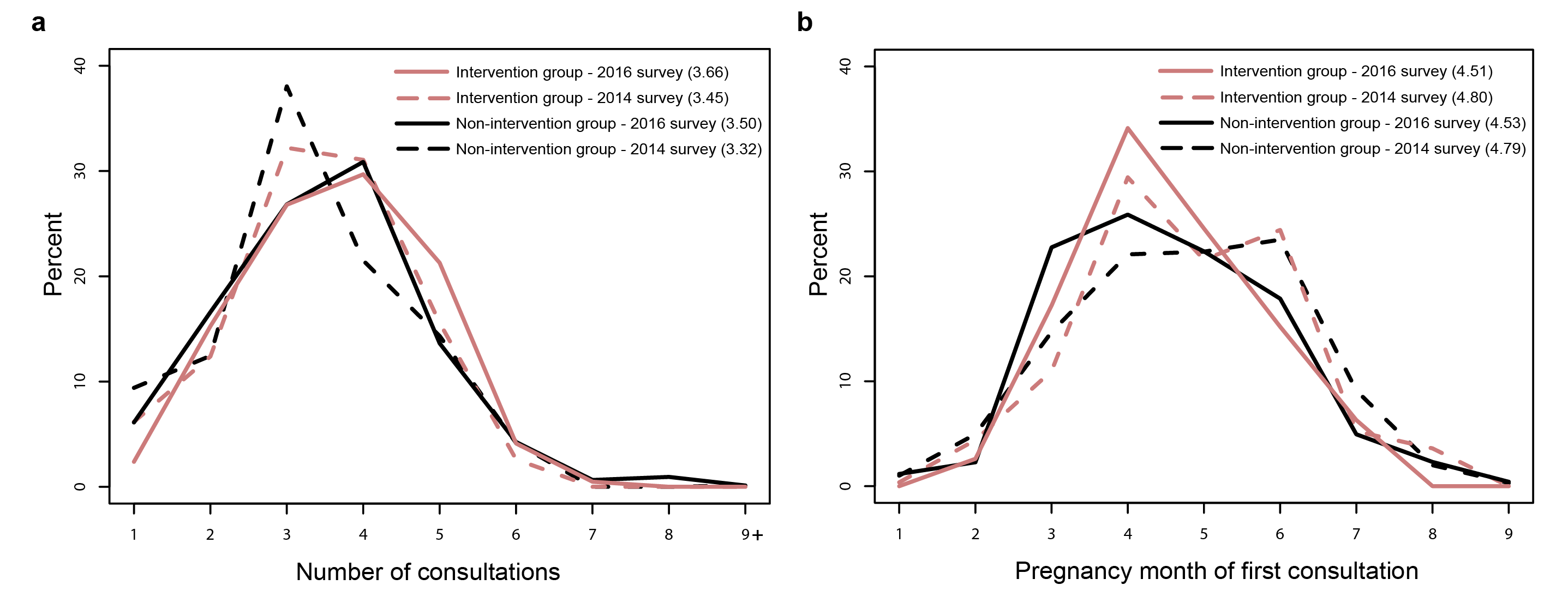


**Appendix 4.** **Additional information on the IHOPE cohort**

In this study, we examined care administered at MoH’s public health facilities (PHF) – which include the district hospital and any of the district’s 13 primary health centers (Centre de Santé de Base 2) – and at community health worker sites (CHW) located throughout the district. We did not consider private pharmacies or clinics in our analysis as they were not part of the PIVOT-MoH intervention. While some private clinics do exist in the Ifanadiana district, they are very limited in the services they provide and account for a marginal proportion of medical visits occurring in the district.

Indicators measured were designed based questions included in the IHOPE surveys and recommendations from WHO guidelines, as defined below. For sick-child care, we followed WHO’s 2014 Integrated Management of Childhood Illness [2]; for antenatal care, we followed WHO’s 2016 Recommendations on Antenatal Care for a Positive Pregnancy Experience [3]; for perinatal care, we followed WHO’s 2016 Standards for Improving Quality of Maternal and Newborn Care in Health Facilities [4]. Although the WHO guidelines had extensive recommendations for each type of care, we focused only on the outcomes that could be evaluated from questions included in the IHOPE surveys.

Definition of indicators assessed in IHOPE survey for child and maternal care, with corresponding WHO guideline recommendations.

| **Indicator** | **Definition** | **WHO Recommendations** |
| --- | --- | --- |
| **Guidelines for sick-child care (<5 years old) based on WHO’s Integrated Management of Childhood Illness (2014)** | | |
| *Children with diarrhea* | | |
| Oral rehydration therapy prescribed at a PHF or CHW | Proportion of children who received oral hydration solution or a fluid recommended by the MoH at a PHF or CHW | Recommended for all children, regardless of severity of symptoms and breastfeeding status |
| Antibiotics prescribed at a PHF or CHW | Proportion of children who received antibiotics at a PHF or CHW (type of antibiotic not specified) | Recommended for children with dysentery (blood in stool) or suspected cholera |
| Homemade-remedy prescribed at a PHF or CHW | Proportion of children who received a homemade remedy (not recommended by the MoH) or herbal medicine at a PHF or CHW | Not recommended |
| Antidiarrheal medication prescribed at a PHF or CHW | Proportion of children who received anti-motility agents (antidiarrheal medication to reduce stool frequency) at a PHF or CHW | Not recommended |
| Zinc supplements prescribed at a PHF or CHW | Proportion of children who received zinc - with oral rehydration therapy kit (ViaSur/HydraZinc) or separately - at a PHF or CHW | Recommended for all children, regardless of severity of symptoms and breastfeeding status |
| *Children with fever* | | |
| NSAIDs/paracetamol prescribed at a PHF or CHW | Proportion of children who received Paracetamol (acetaminophen), Ibuprofen, or other NSAID at a PHF or CHW | Recommended for high fever (38.5 deg Celsius or above) |
| Antibiotics prescribed at a PHF or CHW | Proportion of children who received antibiotics at a PHF or CHW (type of antibiotic not specified) | Recommended for children with identified bacterial cause of fever, regardless of severity of symptoms |
| Malarial rapid diagnostic test administered at a PHF or CHW | Proportion of children whose blood was drawn for a malarial rapid diagnostic test at a PHF or CHW | Recommended for all children living in high malaria risk areas (Ifanadiana district) |
| Antimalarial medication prescribed at a PHF or CHW | Proportion of children who received Sulfadoxine/pyrimethamine (Fansidar), Chloroquine, Amodiaquine, Quinine, other artemisinin-based combination therapies, or other antimalarial medication at a PHF or CHW | Recommended for children who test positive for malaria or who have symptoms of severe febrile illness (stiff neck or general danger signs: vomiting, convulsions, loss of consciousness, or unable to drink/breastfeed) |
| *Children with persistent cough and difficulty breathing* | | |
| Antibiotics prescribed at a PHF or CHW | Proportion of children who received antibiotics at a PHF or CHW (type of antibiotic not specified) | Recommended for children with suspected pneumonia (chest indrawing or fast breathing) or who have symptoms of severe illness (stridor or general danger signs: vomiting, convulsions, loss of consciousness, or unable to drink/breastfeed) |
| NSAIDs/paracetamol prescribed at a PHF or CHW | Proportion of children who received Paracetamol (acetaminophen), Ibuprofen, or other NSAID at a PHF or CHW | Recommended for all children to soothe throat or relieve the cough with safe remedy (type of remedy not specified) |
| Antimalarial medication prescribed at a PHF or CHW | Proportion of children who received Sulfadoxine/pyrimethamine (Fansidar), Chloroquine, Amodiaquine, Quinine, other artemisinin-based combination therapies, or other antimalarial medication at a PHF or CHW | Not recommended, unless child also has symptoms of fever |
| **Guidelines for antenatal care based on WHO’s Recommendations on Antenatal Care for a Positive Pregnancy Experience (2016)** | | |
| 4 or more consultations at a PHF | Proportion of pregnant women who attended at least 4 consultations throughout their pregnancy at a PHF | The 2016 WHO Antenatal Care (ANC) model recommends a minimum of 8 antenatal consultations (1 in the 1st trimester; 2 in the 2nd trimester; 5 in the 3rd trimester). Previous guidelines from the Focused ANC (FANC) model recommended 4 consultations (1 in the 1st trimester; 1 in the 2nd trimester; 2 in the 3rd trimester) |
| 1 or more consultations at a PHF within the first trimester | Proportion of pregnant women who attended at least one consultation during their first trimester of pregnancy at a PHF | The 2016 WHO Antenatal Care (ANC) model recommends the first antenatal consultation to occur anytime within the first 12 weeks (1st trimester) of pregnancy. Previous guidelines from the Focused ANC (FANC) model recommended the first visit occur within the first 8-12 weeks of pregnancy |
| Weight measured at a PHF | Proportion of pregnant women whose weight was measured at least once during any of their antenatal consultations at a PHF | Recommended as part of good clinical practices |
| Blood pressure measured at a PHF | Proportion of pregnant women whose blood pressure was measured at least once during any of their antenatal consultations at a PHF | Recommended as part of good clinical practices |
| Blood sample taken at a PHF | Proportion of pregnant women whose blood was tested at least once during any of their antenatal consultations at a PHF (type of test not specified) | Full blood count testing is the recommended method for diagnosing anemia in pregnancy. In settings where full blood count testing is not available, on-site hemoglobin testing with a haemoglobinometer is recommended |
| Urine sample taken at a PHF | Proportion of pregnant women whose urine was tested at least once during any of their antenatal consultations at a PHF (type of test not specified) | Midstream urine culture is the recommended method for diagnosing asymptomatic bacteriuria (ASB) in pregnancy. In settings where urine culture is not available, on-site midstream urine Gram staining is recommended |
| Counseled about potential pregnancy complications at a PHF | Proportion of pregnant women who received counseling about potential pregnancy complications at least once during any of her antenatal consultations at a PHF | Birth preparedness and complication readiness interventions are recommended to increase the use of skilled care at birth and to increase the timely use of facility care for obstetric and newborn complications |
| **Guidelines for perinatal care based on WHO’s Standards for Improving Quality of Maternal and Newborn Care in Health Facilities (2016)** | | |
| Newborn’s weight measured at a PHF | Proportion of newborn whose weight was measured immediately after delivery at PHF | Recommended to all newborns be weighed at birth to identify low-birth-weight babies (< 2500 g) and provide additional care |
| Newborn breastfed within 1 hr of delivery at a PHF | Proportion of mothers who attempted to breastfeed their newborn within the first  hour of delivery at a PHF | Recommended all mothers be supported to initiate breastfeeding as soon as possible after birth, within the first hour after delivery |
| Newborn exclusively breastfed for first 3 or more days after delivery at a PHF | Proportion of mothers who exclusively breastfed their newborn for at least first 3 days after delivery at a PHF (exclusive breastfeeding for duration of 6 months could not be assessed given that some mothers delivered less than 6 months prior to survey date) | Recommended all babies be exclusively breastfed from birth until 6 months of age |
| Mother’s health checked at a PHF within 6 hrs of delivery at a PHF | Proportion of mothers who received a physical exam by a healthcare professional (physician, nurse, medical assistant, or midwife) within 6 hours of delivery at a PHF | Recommended all postpartum women receive regular assessments (e.g. blood pressure, pulse, temperature) during first 24 hours after delivery |
| Newborn’s health checked at a PHF within 6 hrs of delivery | Proportion of newborn who received a physical exam by a healthcare professional (physician, nurse, medical assistant, or midwife) within 6 hours of delivery at PHF | Recommended all newborns receive a full clinical examination before discharge |

**Appendix 5. Additional information on the SARA survey**

We conducted the WHO Service Availability and Readiness Assessment (SARA) survey, adapted to Malagasy health system context and norms (e.g. number of personnel, list of essential medicines, etc.), over two consecutive years (2014, 2015). The survey was administered only to the 4 PIVOT-supported health centers located in the HSS catchment area. Data collectors were composed of a Monitoring & Evaluation team leader, a paramedic and a logistics coordinator, who were trained on SARA methodology before each survey. Data was collected through observations of the state of the health facilities and testing of equipment as well as interviews of health practitioners. Non-functional medical equipment was recorded as not meeting norms. Medication availability was verified by visual inspection and stockouts were confirmed with the pharmacist.

Indicators measured are defined below. WHO’s 2013 SARA framework includes three main focus areas [5]: 1. General service availability 2. General service readiness and 3. Service-specific readiness. Indicators of general service availability (including health infrastructure and service utilization) were not assessed in this survey because WHO’s national-level targets could not be adapted to district-level measurements. Furthermore, we assess only availability, not readiness, of specific services. For each indicator, we reported the mean proportion score across the four health centers assessed.

Definition of indicators assessed in SARA survey, adapted from WHO to meet MoH standards.

| **Indicator** | **Definition** |
| --- | --- |
| **General service availability:** refers to the physical presence of the delivery of services and encompasses health infrastructure, core health personnel and aspects of service utilization | |
| 1. Health infrastructure | *Indicators not assessed in this survey. WHO’s national-level targets could not be adapted to district-level measurements* |
| 1. Health workforce |  |
| 1. Service utilization |  |
| **General service readiness:** refers to the overall capacity of health facilities to provide general health services | |
| 1. Personnel level | Available proportion of 5 essential personnel positions (%): 1 physician, 1 nurse, 1 midwife, 1 guard, 1 pharmacist |
| 1. Basic amenities | Available proportion of 5 basic amenities items (%): power, improved water source, adequate sanitation facilities, communication equipment, sufficient workspace |
| 1. Basic equipment | Available proportion of 6 basic equipment items (%): adult scale, child scale, thermometer, stethoscope, blood pressure apparatus, examination table |
| 1. Standard precautions for infection prevention | *Indicator not assessed in this survey* |
| 1. Diagnostic capacity | *Indicator not assessed in this survey* |
| 1. Essential medicines | Available proportion of 15 essential medicines (%): aminophylline, amoxicillin, benzylpenicillin, captopril, chlorpheniramine, co-trimoxazole, iron-folic acid, gentamicin, hydrochlorothiazide, ibuprofen, metoclopramide, metronidazole, paracetamol, phenobarbital, oral rehydration therapy/zinc packets |
| **Specific service availability/readiness***: refers to the ability of health facilities to offer a specific service, and the capacity to provide that service measured through consideration of tracer items that include trained staff, guidelines, equipment, diagnostic capacity, and medicines and commodities | |
| 1. Preventative services | Available proportion of 4 preventative services available (%): antenatal care, postnatal care, family planning, childhood immunization |
| 1. Therapeutic services | Available proportion of 5 therapeutic services (%): outpatient care, inpatient care, obstetric care, hospitalization capabilities, treatment capabilities |
| 1. Health promotion and administration services | Available proportion of 3 health promotion and administration services (%): health promotion, community outreach, health management information system |
| 1. Complementary services | Available proportion of 3 complementary services (%): malnutrition care, tuberculosis care, IMCI guidelines implementation |
| * This survey assessed only for availability of specific services; the WHO SARA survey assesses for availability and readiness. | |

**References**

1. Kruk ME, Gage AD, Arsenault C, Jordan K, Leslie HH, Roder-DeWan S, et al. High-quality health systems in the Sustainable Development Goals era: time for a revolution. The Lancet Global health. 2018;6(11):e1196-e252.

2. The World Health Organization. Integrated Management of Childhood Illness - Chart Booklet. 2014.

3. The World Health Organization. WHO recommendations on antenatal care for a positive pregnancy experience. 2016.

4. The World Health Organization. Standards for improving quality of maternal and newborn care in health facilities. 2016.

5. The World Health Organization. Service Availability and Readiness Assessment (SARA): An annual monitoring system for service delivery - Reference Manual. 2013.
