# Supplementary material for: Assessing trends in the content of maternal and child care following a health system strengthening initiative in rural Madagascar: A longitudinal cohort study
Source: PLoS Med. 2019 Aug 20;16(8):e1002869. doi: 10.1371/journal.pmed.1002869 (PMC6701767; doi:10.1371/journal.pmed.1002869)
Supplement: S1 TIDIER Checklist — HSS, health system strengthening; TIDieR, Template for Intervention Description and Replication. (DOCX) [file pmed.1002869.s002.docx]

**Assessing trends in the content of maternal and child care following a health system strengthening initiative in rural Madagascar: a longitudinal cohort study**

**S1 Checklist - Summary of the HSS Intervention carried out by PIVOT in Ifanadiana District between 2014 and 2016,**

**based upon the Template for Intervention Description and Replication (TIDieR) checklist**

| **1. BRIEF NAME** |
| --- |
| Integrated health system strengthening initiative in Ifanadiana district, Madagascar |
| **2. WHY** |
| ***GOAL/RATIONALE:*** To create a model public health district with universal access to care aiming for broad-based population health impact on mortality. Based upon the World Health Organization’s building blocks of Health System Strengthening: 1) service delivery; 2) health workforce; 3) health information systems; 4) medicines and supplies; 5) financing; 6) leadership. |
| **WHAT** |
| **3. *MATERIALS*** (by level of care and above enumerated building blocks)  **At district hospital level**  (1) *Service delivery:* Overall infrastructure upgrades and outfitting for service delivery, bringing inpatient bed capacity from 25 to 40, upgrades to waste management system, specific renovations of the emergency and triage department, pediatric unit, inpatient ward, isolation ward, and laboratory; support to specific service delivery, including emergency care and provision of a network of 3 ambulances with 24/7 coverage and 9 PIVOT paramedics for referrals; maternal and obstetrical care; laboratory service (staffing and equipment upgrades meant tuberculosis could be diagnosed in the district again); social support for vulnerable patients.  (2) *Health workforce:* Staffing of health workers to reach MoH norms through joint PIVOT-MoH hires of 7 clinicians, including a trauma surgeon and an anesthesiologist; staffing supplemented further with fulltime presence of 2 PIVOT doctors and 2 nurses by end of 2016; non-clinical PIVOT staff including a team of 4 social workers, support staff (janitors, guards, etc.), a laboratory technician, and a radiology technician; ongoing mentorship and frequent trainings of medical staff in key clinical areas, such as emergency medicine and postoperative care.  (3) *Health information systems:* Creation of a hospital-based M&E team to follow progress of activities and improve quality of HMIS data; implementation of a system for baseline and follow-up facility readiness surveys.  (4) *Medicines and supplies:* Supply chain management and reduction of stock-outs, initially through frequent donations which evolved into a reimbursement program paired with pharmacy management training; provision of medical and non-medical equipment for service delivery, including full laboratory capacity.  (5) *Financing:* Cost of outpatient and inpatient care fully covered for patients referred by district-wide health centers and self-referred patients who necessitated urgent inpatient care (more than 5,300 patients by the end of 2016); cost of referral to and care at higher levels of care (e.g. university hospital) fully covered for services not available at district hospital.  (6) *Leadership:* Creation of a joint PIVOT-MoH executive committee for hospital management and transparency; creation of sub committees for specific projects such as infection control or quality of care.  **At health center level**  (1) *Service delivery:* Overall infrastructure renovations and/or extensions for service delivery at the 4 target health centers, including ensuring electricity, water, waste management/sterilization capacity, proper pharmacy conditions; provision of medical and non-medical equipment, including beds, armoires, furniture; support to launching specific service delivery of Integrated Management of Childhood Illnesses and malnutrition protocols for every child under 5 attending the health center; ensuring timely referrals and emergency care.  (2) *Health workforce:* Staffing through joint MoH-PIVOT hires to bring all 13 primary care health centers up to MoH norms (1 doctor, 1 nurse, 1 midwife, 1 dispenser, 1 support staff at each facility); at target health facilities, hiring exceeded norms and PIVOT clinicians were permanently present (~2 clinicians per health center) to implement service delivery protocols (IMCI, malnutrition); by end of 2016, there were 42 joint MoH-PIVOT hires, comprising 58% of total MoH health center staff in the district; trainings for medical staff (some district-wide) such as obstetrical and neonatal care; ongoing supervision and mentorship in target centers for IMCI and malnutrition.  (3) *Health information systems:* Joint MoH-PIVOT training and supervision to improve HMIS data quality (district-wide); implementation of system for baseline and follow-up of facility readiness surveys.  (4) *Medicines and supplies:* Supply chain management and reduction of stock-outs, initially through frequent donations which evolved into a reimbursement program paired with pharmacy management training.  (5) *Financing:* Essential medicines and consumables provided free of charge to all patients (more than 60,000 patients between October 2014 launch and end of 2016); full details of this program are available at Garchitorena et al. (2017).  (6) *Leadership:* Close collaboration with district health managers for the planning and implementation of activities.  **At community level**  (1) *Service delivery:* Construction of 21 community health posts; specific service delivery in Integrated Management of Childhood Illnesses and malnutrition protocols for every child under 5, community sensitization and mass testing, urgent care, and mobile clinics with direct care provision by PIVOT clinicians every other month.  (2) *Health workforce:* Training, coaching and monthly supervision of ~50 community health workers by mobile teams of trained nurses by the end of 2016 (6 nurses for ~50 health workers); community IMCI training provided for CHWs in all 13 communes.  (3) *Health information systems:* Joint MoH-PIVOT training to improve HMIS data quality on community health.  (4) *Medicines and supplies:* Monthly provision and follow-up of MNCH medicine stocks, including malaria diagnosis and treatment, oral rehydration salts, NSAIDS, antibiotics and zinc.  (5) *Financing:* cost of MNCH medicine stocks fully covered; financial and non-financial incentives to community health workers and local leadership.  (6) *Leadership:* Community engagement and participation (e.g. community health posts are built by the community, with PIVOT support for roofing, painting, furniture and equipment).  **4. *PROCEDURES***  All interventions were aimed at fulfilling existing Madagascar Ministry of Health protocols and standards. |
| **5. WHO PROVIDED** |
| **At district hospital level**  Ministry of Health clinicians provided the majority of service delivery. PIVOT clinicians are integrated in the hospital staff and provide direct care as any other clinician during external consultations and clinical rounds, but also carry out frequent training. Non-clinical PIVOT staff provided social support to vulnerable patients, helped manage the patient circuit to benefit from fee exemptions (registration, validation).  **At health center level**  Ministry of Health doctors and nurses provided the majority of service delivery. By MoH norms each health center (CSB2) should have 1 doctor, 1 nurse, 1 midwife, 1 dispenser, and 1 support staff. Additional PIVOT clinicians (~2 per health facility) provided some direct care, especially for the implementation of malnutrition and IMCI protocols, but focused mostly on training and supervision.  **At community level**  Two community health workers per fokontany (a cluster of villages, lowest administrative unit) provided basic MNCH care, supervised monthly by the clinicians of their respective health center. PIVOT mobile teams of nurses provided on-site mentoring and supervision of CHWs every two months. They also provided direct care at community level during on-site supervisions for fokontany located >10 kilometers from a health center. |
| **6. HOW (modes of delivery)** |
| PIVOT employees worked in partnership with existing networks of MoH clinicians and community health workers within existing public health facilities. Wherever possible, such as in the case of supply chain management, leadership, and financing- the intervention deliberately avoids the creation of parallel systems of care. |
| **7. WHERE** |
| **At district hospital level**  The initial PIVOT catchment area comprised the only district hospital, located in Ifanadiana city. Most referrals to higher levels of care (tertiary) were sent to the university hospital in Fianarantsoa (2h away by car), and some to specialized facilities in Antananarivo (capital, ~1 day by car).  **At health center level**  Full health center activities were implemented in the health centers of the four communes closest to the hospital on the district’s sole paved road (i.e. Ranomafana, Kelilalina, Ifanadiana, Tsaratanana); all 13 health centers in the district received trainings, staffing support to reach MoH norms, and some access to the referral network (limited by accessibility).  **At community level**  By 2016, community activities had been rolled out in 21 fokontany from two out of the four communes within the PIVOT catchment area (out of a total of 195 total fokontany in the district). |
| **8. WHEN AND HOW MUCH** |
| All interventions were progressively rolled out during the study period.  The earliest intervention activities implemented (starting in April-May 2014, at the beginning of the study period) included the ambulance network, staffing of health centers and district hospital, and provision of medical equipment.  The renovation of health centers also began in April-May 2014 but the date of completion varied for each health facility.  The renovation of the emergency and triage unit and pediatric guard at the district hospital were completed by early 2016.  Removal of user fees at health centers and hospital began in October of 2014.  Implementation of IMCI and malnutrition protocols at all health centers began in October 2015.  Community-level activities began in November 2015. |
| **9. TAILORING** |
| N/A |
| **10. MODIFICATIONS** |
| N/A; The intervention is progressively being implemented, as explained in section 8 (when and how much) |
| **HOW WELL** |
| N/A; The aim of this study was to study the evolution of intervention coverage in Ifanadiana District. Full details of the impact assessment are available in the main text. |
