# Supplementary material for: Assessing trends in the content of maternal and child care following a health system strengthening initiative in rural Madagascar: A longitudinal cohort study
Source: PLoS Med. 2019 Aug 20;16(8):e1002869. doi: 10.1371/journal.pmed.1002869 (PMC6701767; doi:10.1371/journal.pmed.1002869)
Supplement: S1 STROBE Checklist — STROBE, Strengthening the Reporting of Observational Studies in Epidemiology. (DOCX) [file pmed.1002869.s003.docx]

**Assessing trends in the content of maternal and child care following a health system strengthening initiative in rural Madagascar: a longitudinal cohort study**

**S2 Checklist - STROBE Statement—Checklist of items that should be included in reports of *cohort studies***

|  | Item No | Recommendation | Page No |
| --- | --- | --- | --- |
| **Title and abstract** | 1 | (*a*) Indicate the study’s design with a commonly used term in the title or the abstract | a) Title: *Assessing trends in the content of maternal and child care following a health system strengthening initiative in rural Madagascar: a longitudinal cohort study* |
|  |  | (*b*) Provide in the abstract an informative and balanced summary of what was done and what was found | b) The abstract provides this information |
| Introduction | | | |
| Background/rationale | 2 | Explain the scientific background and rationale for the investigation being reported | In paragraphs 1-4 of the Introduction section |
| Objectives | 3 | State specific objectives, including any prespecified hypotheses | In paragraphs 5-6 of the Introduction section |
| Methods | | | |
| Study design | 4 | Present key elements of study design early in the paper | In paragraph 3 of the Methods section (Data collection subsection) |
| Setting | 5 | Describe the setting, locations, and relevant dates, including periods of recruitment, exposure, follow-up, and data collection | In paragraphs 1-2 of the Methods section (Study intervention subsection), Figure 1, and S1 TIDIER Checklist |
| Participants | 6 | (*a*) Give the eligibility criteria, and the sources and methods of selection of participants. Describe methods of follow-up | a) In paragraph 3 of the Methods section (Data collection subsection) |
|  |  | (*b*) For matched studies, give matching criteria and number of exposed and unexposed | b) The non-intervention comparison group is described in paragraph 9 of the Methods section (Data analysis subsection) |
| Variables | 7 | Clearly define all outcomes, exposures, predictors, potential confounders, and effect modifiers. Give diagnostic criteria, if applicable | In paragraphs 6-7 of the Methods section (Outcomes subsection) and elaborated in paragraph 7-8 of the Discussion section |
| Data sources/ measurement | 8* | For each variable of interest, give sources of data and details of methods of assessment (measurement). Describe comparability of assessment methods if there is more than one group | In paragraph 6-7, 9-10 of the Methods section (Outcomes and Data analysis subsections) |
| Bias | 9 | Describe any efforts to address potential sources of bias | In paragraph 10 of the Methods section (Data analysis subsection) and elaborated in paragraphs 7-8 of the Discussion section |
| Study size | 10 | Explain how the study size was arrived at | In paragraph 3 of the Methods section (Data collection subsection) |
| Quantitative variables | 11 | Explain how quantitative variables were handled in the analyses. If applicable, describe which groupings were chosen and why | In paragraphs 6-7 of the Methods section (Outcomes subsection) |
| Statistical methods | 12 | (*a*) Describe all statistical methods, including those used to control for confounding | a-c) In paragraphs 9-10 of the Methods section (Data analysis subsection) |
|  |  | (*b*) Describe any methods used to examine subgroups and interactions | d) In paragraph 3 of the Methods section (Data collection subsection)  e) No sensitivity analyses were done |
|  |  | (*c*) Explain how missing data were addressed |  |
|  |  | (*d*) If applicable, explain how loss to follow-up was addressed |  |
|  |  | (*e*) Describe any sensitivity analyses |  |
| Results | | |  |
| Participants | 13* | (a) Report numbers of individuals at each stage of study—eg numbers potentially eligible, examined for eligibility, confirmed eligible, included in the study, completing follow-up, and analysed | a-b) In paragraph 1 of the Results section and Table 1  c) Flow diagram was not used (available in Miller AC, Garchitorena A, Rabeza V, Randriamanambintsoa M, Rahaniraka Razanadrakato HT, Cordier L, et al. Cohort Profile: Ifanadiana Health Outcomes and Prosperity longitudinal Evaluation (IHOPE). International journal of epidemiology. 2018.) |
|  |  | (b) Give reasons for non-participation at each stage |  |
|  |  | (c) Consider use of a flow diagram |  |
| Descriptive data | 14* | (a) Give characteristics of study participants (eg demographic, clinical, social) and information on exposures and potential confounders | a-c) In paragraph 1 of the Results section and Table 1. Missing data information in S2 Table |
|  |  | (b) Indicate number of participants with missing data for each variable of interest |  |
|  |  | (c) Summarise follow-up time (eg, average and total amount) |  |
| Outcome data | 15* | Report numbers of outcome events or summary measures over time | In Tables 2-3, Figures 2-3, and S1 Appendix |

| Main results | 16 | (*a*) Give unadjusted estimates and, if applicable, confounder-adjusted estimates and their precision (eg, 95% confidence interval). Make clear which confounders were adjusted for and why they were included | a) In paragraphs 2-4 of the Results section, Tables 2-3, and Figures 2-3, S1 Appendix, and S1 Table  b) In Table 1 for demographic characteristics; in Table 3 and S1 Appendix for antenatal care seeking behaviour  c) Percent relative change provided with rates for each year in parentheses throughout the Results section |
| --- | --- | --- | --- |
|  |  | (*b*) Report category boundaries when continuous variables were categorized |  |
|  |  | (*c*) If relevant, consider translating estimates of relative risk into absolute risk for a meaningful time period |  |
| Other analyses | 17 | Report other analyses done—eg analyses of subgroups and interactions, and sensitivity analyses | Service availability and readiness analysis reported in paragraph 5 and Table 4 of the Results section |
| Discussion | | | |
| Key results | 18 | Summarise key results with reference to study objectives | In paragraphs 1-3, 6 of the Discussion section |
| Limitations | 19 | Discuss limitations of the study, taking into account sources of potential bias or imprecision. Discuss both direction and magnitude of any potential bias | In paragraphs 8-9 of the Discussion section |
| Interpretation | 20 | Give a cautious overall interpretation of results considering objectives, limitations, multiplicity of analyses, results from similar studies, and other relevant evidence | In paragraph 1 of the Conclusion section |
| Generalisability | 21 | Discuss the generalisability (external validity) of the study results | In paragraphs 4-7 of the Discussion section |
| Other information | | | |
| Funding | 22 | Give the source of funding and the role of the funders for the present study and, if applicable, for the original study on which the present article is based | Specified in the PLOS submission form: The authors received no external source of funding for this work. The IHOPE and SARA studies were funded by the non-governmental organization PIVOT and the Herrnstein Family Foundation. The funders had no role in study design, data collection and analysis, decision to publish, or preparation of the manuscript. |

*Give information separately for exposed and unexposed groups.

**Note:** An Explanation and Elaboration article discusses each checklist item and gives methodological background and published examples of transparent reporting. The STROBE checklist is best used in conjunction with this article (freely available on the Web sites of PLoS Medicine at http://www.plosmedicine.org/, Annals of Internal Medicine at http://www.annals.org/, and Epidemiology at http://www.epidem.com/). Information on the STROBE Initiative is available at http://www.strobe-statement.org.
